# Synthetic Lethality through the Lens of Medicinal Chemistry

**DOI:** 10.1021/acs.jmedchem.0c00766

**Published:** 2020-11-02

**Authors:** Samuel H. Myers, Jose Antonio Ortega, Andrea Cavalli

**Affiliations:** † Computational & Chemical Biology, Istituto Italiano di Tecnologia, 16163 Genova, Italy; ‡ Department of Pharmacy and Biotechnology, University of Bologna, 40126 Bologna, Italy

## Abstract

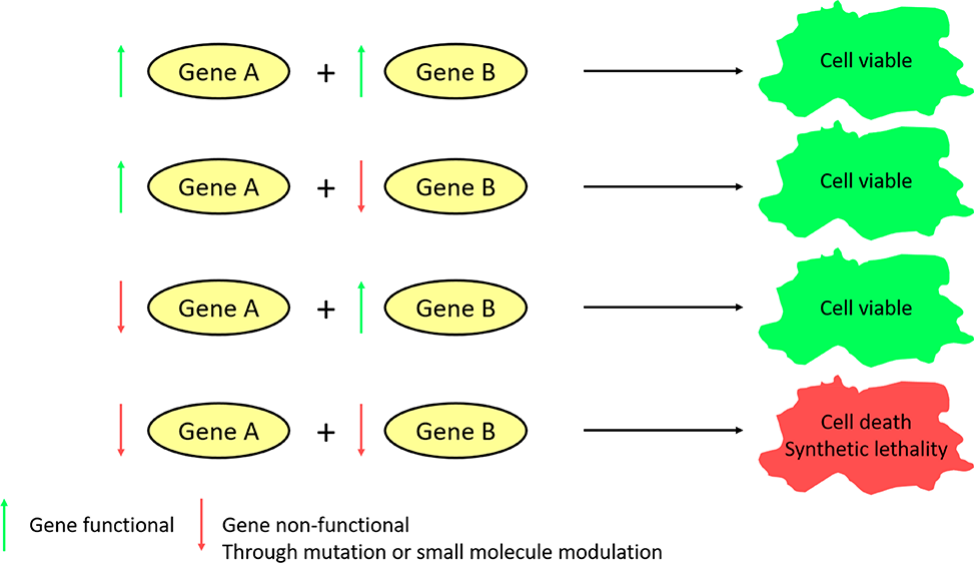

Personalized medicine and therapies represent the goal of modern medicine, as drug discovery strives to move away from one-cure-for-all and makes use of the various targets and biomarkers within differing disease areas. This approach, especially in oncology, is often undermined when the cells make use of alternative survival pathways. As such, acquired resistance is unfortunately common. In order to combat this phenomenon, synthetic lethality is being investigated, making use of existing genetic fragilities within the cancer cell. This Perspective highlights exciting targets within synthetic lethality, (PARP, ATR, ATM, DNA-PKcs, WEE1, CDK12, RAD51, RAD52, and PD-1) and discusses the medicinal chemistry programs being used to interrogate them, the challenges these programs face, and what the future holds for this promising field.

## 1 Introduction

It is commonly known that cancer is a disease of the genome, wherein errors in DNA replication and repair cause failures in cell function, while the mechanisms that would normally deal with these faulty cells are also compromised, resulting in cancer cell survival and ultimately proliferation. Before the advent of the human genome project in 2003, cancer treatments were mainly radiotherapy and nonspecific chemotherapy: two treatment methods that are famed for their lack of specificity and severe side effects. The human genome project has aided the route toward personalized medicine, wherein unique biomarkers in individuals can be detected and treated with tailor-made therapeutics for that specific cancer. There have been numerous success stories, primarily where “oncogene addiction” occurs, in which a particular subtype of cancer is over-reliant for survival on a particular oncogene, and therefore inhibition of this oncogene through small molecules or antibodies causes selected cell death. Examples include imatinib, which targets BCR-ABL that has extended the median survival of patients with chronic myelogenous leukemia to greater than 10 years.^1^ Another success story can be seen in melanoma, where treatment with the BRAF inhibitor vemurafenib has caused melanoma to change from a mostly untreatable disease to one where over 50% of patients show a meaningful clinical response.^2^ However, this trend toward precision-based personalized medicine has fallen out of fashion in the cancer drug discovery community. First, not all cancers show a simple oncogene addiction, and their survival mechanisms are far more complex; also, certain oncogenes have proved undruggable by small molecules and antibodies.^3^ Furthermore, even with initial success, issues of resistance and poor mechanistic understanding have hampered efforts, resulting in oncology still reliant on radiotherapy and nonspecific chemotherapeutics. Attention has turned toward synthetic lethality, exploiting hampered DNA repair mechanisms in cancer and allowing access to previously undruggable targets by indirect inhibition.^4^

Synthetic lethality (SL) was first discovered in the fruit fly (*Drosophila pseudoobscura*) in 1946 ^5^ and can be defined as the relationship that can occur between two genes where either one functioning maintains viability of the cell; however upon dysfunction of both genes, the cell becomes unviable.^6^ In its simplest and most desirable application, this would result in the selective killing of cells that rely on the mechanisms driven by these two genes, i.e., cancer cells, while leaving the healthy cells alive. One synthetically lethal pair, PARP (poly (ADP-ribose) polymerases) and BRCA1/2, are now exploited in standard of care treatments;^7^ however this was 6 years ago, and still no other SL-based drugs have gained regulatory approval.

Within the field of synthetic lethality there are numerous examples of synthetically lethal gene pairs; therefore in this Perspective we select prominent and interesting examples to use as case studies for the field. Within these sections the targets typical mode of function is described, followed by the proposed mechanisms of synthetic lethality. Subsequently, medicinal chemistry programs that have produced molecules with potential to cause “small molecule-induced synthetic lethality” are discussed, including compounds in early stage, preclinical, and clinical studies. The review ultimately concludes with the challenges facing such small molecules and the drugging of their targets.

## 2 PARP and BRCA2

**Figure 1:**
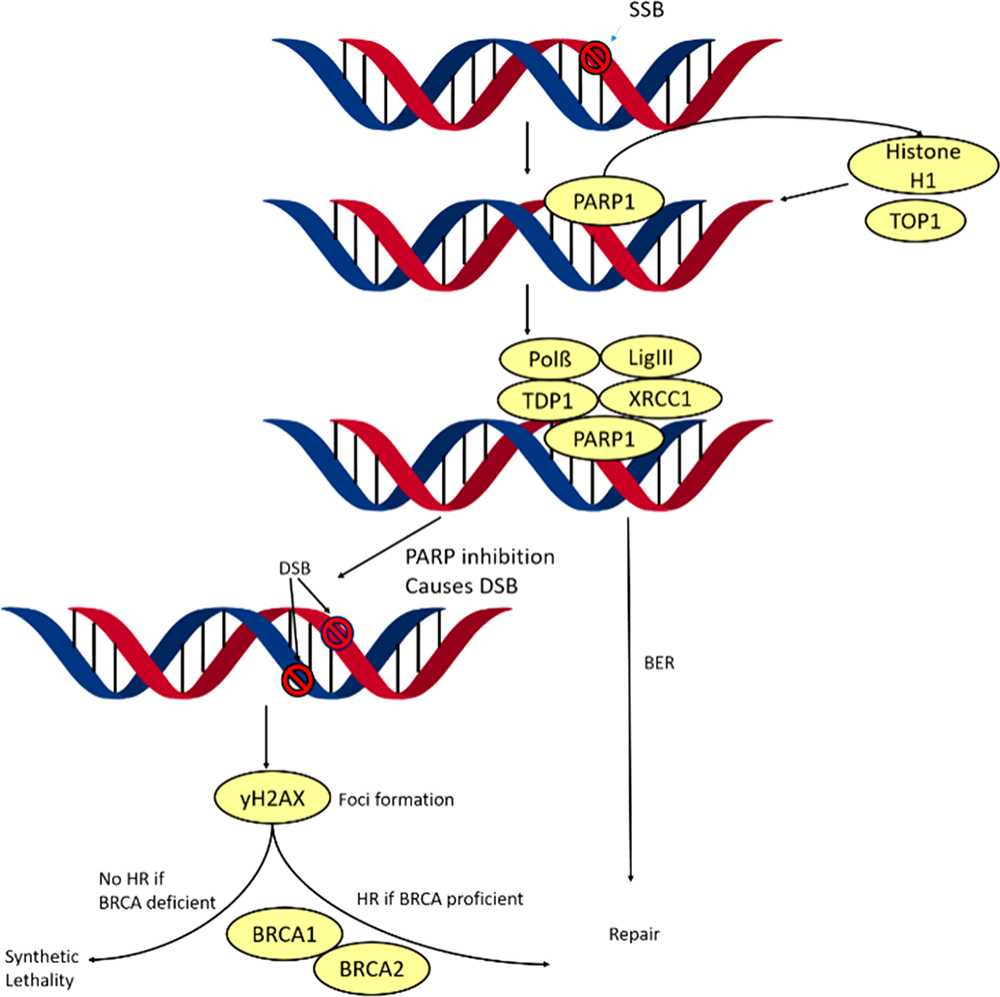
Schematic representation of PARP’s role in SSB and DSB repair. Upon formation of a SSB, PARP1 and other acceptor proteins (histone H1, TOP1, and others) are recruited and attached to the lesion site. This recruits other factors involved in DNA repair (Polβ, LigIII, TDP1, and XRCC1) which, through BER, repair the lesion. If PARP is inhibited, the SSB becomes a DSB which causes γH2AX foci formation, which in the presence of BRCA1/2 triggers HR which repairs the DSB. In the absence of BRCA this break becomes synthetically lethal. Adapted from Clinical Cancer Research, Copyright 2010, Vol. 16, Issue (18), , Page 4532, Christophe E. Redon, Asako J. Nakamura, Yong-Wei Zhang, Jiuping (Jay) Ji, William M. Bonner, Robert J. Kinders, Ralph E. Parchment, James H. Doroshow, Yves Pommier, Histone γH2AX and Poly(ADP-Ribose) as Clinical Pharmacodynamic Biomarkers2082314610.1158/1078-0432.CCR-10-0523PMC2940983,^22^ with permission from AACR.

PARP is a family of enzymes containing 17 members,^8^ of which 15 have been shown to catalyze the transfer ADP-ribose to target proteins.^9,10^ PARP1 and PARP2 play important roles in DNA repair, making them an attractive target for oncology. PARP’s role in DNA repair was first discovered when it was observed that there was a correlation between high amounts of DNA lesions and increased PARP concentration.^11^ PARP is involved in the repair of single-stranded breaks (SSBs), through base excision repair (BER) (Figure 1), where it forms a part of the BER complex.^12^ In addition to this, PARP has been observed to play a role in nucleotide excision repair (NER). This and BER are both mechanisms for DNA repair.^13^ PARP has been observed in cell-free systems to bind tightly to broken DNA, and after auto-poly-ADP-ribosylation, it allows repair enzymes to access the DNA in order to initiate repair.^14,15^

BRCA2 plays an important role in repairing double-stranded breaks (DSBs), as part of the homologous recombination (HR) pathway, and has been reported to be synthetically lethal when disrupted in combination with PARP,^16^ which as stated previously is involved in the repairing of SSB. This relationship was first discovered in 2005,^17,18^ and since this date, numerous papers have been published discussing the mechanism behind this.^19−21^ In the case of BRCA2 and PARP, in patients with defective BRCA2, PARP inhibitors can be administered to induce synthetic lethality.

Numerous cancers have defects in HR: such tumors include ovarian (50%) breast, prostate, and pancreatic cancers (all 10–20%).^23^ This is thought to aid cancer initiation and progression, with the hypothesis being that this fault in HR will cause more DNA breaks, resulting in more mutations. Due to cancer cell survival mechanisms, these DNA breaks may not necessarily be fatal to the cell and in fact may allow for more favorable conditions for cell survival.^24^ Due to the high proportion of cancers that express these HR defects, they are prime candidates for treatment with PARPi. Three main mechanisms have been hypothesized to exploit the synthetically lethal relationship between PARP and BRCA2. The first begins with a PARP inhibitor (PARPi) blocking BER, thus causing the conversion of an SSB to a DSB that BRCA2 would normally fix. If the tumor is genetically deficient in BRCA2, it will not be able to perform HR and therefore cause irreparable DNA damage, leading to cell death.^23^ In the second mechanism, the PARPi binds to the PARP1 enzyme on the chromatin, thus trapping it in mechanism referred to as PARP trapping; this causes a lesion that must be repaired which, due to compromised HR, is not possible and causes cell death. In the third mechanism, DSBs are resected during the S phase; however, since HR is defective, the alternative microhomology-mediated end joining (MMEJ) pathway attempts to repair the break. Since PARP1 is inhibited by the PARPi, it can no longer recruit POLQ, therefore blocking this pathway and triggering cell death;^25^ the same effect is thought to be achieved through POLQ dysregulation.^26^

**Figure 2:**
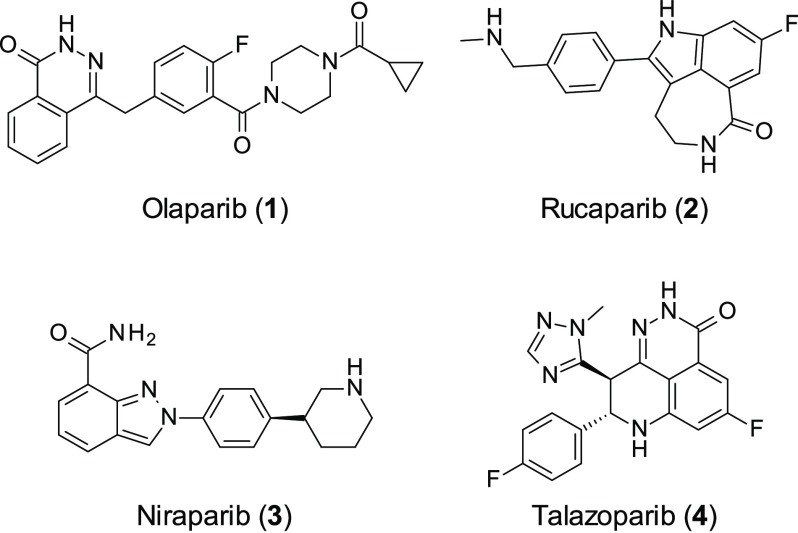
Approved PARP inhibitors.

PARP inhibitors demonstrate the first major success in the field of synthetic lethality and became the first evidence of modulating SL in the clinic when, in 2014, olaparib (**1**, Figure 2) became the first PARPi approved by the FDA. This was approved for the treatment of ovarian cancer, following a phase II trial in which 34% of patients exhibiting BRCA1/2 mutations showed compelling response rates. The patients had been given at least three rounds of chemotherapy previously and were then treated with **1** as a single agent.^27^ It was seen in this trial and subsequent ones that platinum-sensitive cancers showed a better overall response rate to PARPi compared to platinum-resistant cancers.^28,29^ Despite this, **1** has shown some success in platinum-resistant cancers in patients with BRCA1/2 mutations.^28,30^ This diversifies it from the other approved PARPi’s. **1** was approved in tablet and capsule form for the treatment of advanced ovarian cancer, where the patient had received at least three rounds of prior chemotherapy.

The second PARPi to be approved was rucaparib (**2**, Figure 2) in 2016, which demonstrated similar effects to **1** in phase II trials;^31,32^ however, the side effects between the two drugs are slightly different, affecting treatment choice. **2** was approved for use in patients with advanced ovarian cancer with deficient BRCA1/2 (either germline or somatic), who had received at least two treatments of chemotherapy in the past. The third FDA approved PARPi for the treatment of advanced ovarian cancer is niraparib (**3**, Figure 2), and in 2017, it was approved for patients with recurrent epithelial ovarian, fallopian tube, or primary peritoneal cancer in platinum-sensitive tumors.^33^

PARPi’s have also been approved for the treatment of breast cancer. This was achieved through two phase III trials, studying **1**(34) and talazoparib (**4**, Figure 2)^35^ in comparison to the clinicians’ choice of chemotherapy in patients with human epidermal growth factor receptor 2 (HER-2) negative breast cancer who had received chemotherapy previously and have seen no progression on the platinum-based therapy. PARP inhibition showed an increase in progress-free survival in comparison to the platinum therapies. The final PARPi to gain regulatory approval was **4** in 2018, which is orally administered to adults with deleterious/suspected deleterious germline BRCA-mutated, (HER2)-negative, locally advanced, or metastatic breast cancer;^36^**1** was also approved for the same disease area. Furthermore, **1** was also approved for the treatment of pancreatic cancer as a first line maintenance treatment in cancers with a loss of function of BRCA2, following a double-blind placebo-controlled multicenter trial on 154 patients.^37^ The median progress-free survival of the participants increased from 3.8 months in the placebo to 7.4 months with **1**. Although this is a small increase, any progress in this particularly challenging disease area should be applauded.

Recently (May 2020), **2**, followed by **1**, gained FDA approval for the treatment of germline and/or somatic BRCA-mutated metastatic castration-resistant prostate cancer. **2** was approved as a result of Triton 2, an ongoing multicenter, single arm clinical trial in 115 patients with BRCA-mutated (germline and/or somatic) mCRPC who had been treated with androgen receptor-directed therapy and taxane-based chemotherapy. **2** showed an objective response rate of 44% in 62 patients with measurable disease; 56% of these showed a duration of response greater than 6 months.^38^**1** was approved on the basis of a 2019 phase III trial of 387 men, where **1** increased progression-free survival from 3.6 months to 7.4 months. **1** also decreased the risk of disease progression or death to a median of 5.8 months vs 3.5 months for standard of care treatment.^39^

The field of PARP inhibition has been covered extensively by the literature; last year, an excellent review summarized the field of PARP inhibitors from a medicinal chemistry perspective, taking advantage of the extensive knowledge of the PARP protein to highlight binding motifs and structural similarities between the various generations of PARP inhibitors.^40^ The majority of inhibitors discussed in this review could theoretically be applied to inducing the SL phenotype; the field is extensively covered in the review by Jain et al., in which 37 different inhibitors are discussed with the majority of these being discovered after the approval of **1**.

PARPi resistance can develop in cancer cells showing the BRCA2 mutation, occurring in one of two ways: the cell either finds a way to re-establish HR repair or repairs the break through alternative means, and both are covered in great detail in the review by D’Andrea.^23^ These mutations are relevant in a clinical setting, as 46% of platinum-resistant serious grade ovarian cancers showed a mutation that resulted in the restoration of the BRCA2 pathway. This is achieved either through genetic events that cancel the loss of function of the BRCA2 mutation or through genetic inversion of the mutation, resulting in restoration of the wild-type (WT) BRCA2 protein. These resistance mechanisms demonstrate the continuous need for evolution of understanding of PARP inhibition, and the BRCA1/2 pathway. Examples of how increased understanding of the PARP1/BRCA2 pathway could bypass resistance are shown later in this text, as these resistance mechanisms rely on the restoration of BRCA2 function. Through an increased understanding of the relationship between BRCA2 and RAD51, it has been proposed that PARP1 inhibitor efficacy could be restored through interruption of the BRCA2/RAD51 interaction. Therefore, given the crossover between many DNA repair mechanism modulating targets, it is possible that other key interactions for BRCA2 could be elucidated as understanding of these pathways increases, providing new potential SL-tools to restore PARPi activity.

## 3 ATR, ATM, and DNA-PKcs Inhibitors

ATM and DNA-PKcs exhibit complementary functions in DNA damage repair, notably HR and nonhomologous end joining (NHEJ). The co-dependent nature of these two kinases is evident from the inactivation of these two kinases, causing embryonic lethality.^41^ A loss of function through either genetic malfunction or chemical modulation can cause synthetic lethality within cells. The inhibition of both targets causes an accumulation of DSB, which in turn causes an increase CTBP interacting protein (CtIP) mediated resection, thus forming large single-stranded DNA (ssDNA) tracts, followed by the triggering of apoptosis through the ATR/CHK1 pathway.^42^ This has been achieved with inhibition of DNA-PKcs as a monotherapy in ATM-defective lymphomas.^43^ In addition to ATM, inhibition of DNA-PKcs has been shown to be synthetically lethal in cells in a number of other targets that play a key role in the HR process: BRCA1, BRCA2, CHK2, Rad50, PTIP, and PAXIP.^44^ The inactivation of ATM and DNA-PKcs has been seen to be more effective in BRCA1/2 deficient cells.^45^ The link between ATM and BRCA1/2 is not well understood: it has been hypothesized that rather than being dependent on BRCA1/2, ATM is dependent on certain genetic changes this genotype causes.^46^ Additionally, it has been seen that deficiency of either ATM or DNA-PKcs causes a sensitization to DNA-damaging agents such as topoisomerase I and II poisons or DNA alkylating agents.^47^ It has been proposed that the observed SL mechanism is not due to a failing of the DNA repair mechanism; this would differentiate this pathway somewhat from other SL pathways. Lastly, ATM has been shown to be synthetically lethal with the kinases MAPK and MEK1/2,^48^ with ATM playing a role in prosurvival pathways, noticeably the AKT/mTOR pathway: when MAPK or MEK1/2 is inhibited, if ATM is also impaired, this pathway is not open and therefore cell death occurs. This is important, as MEK inhibitors have already gained regulatory approval.^49^

As shown by its complementary functions with ATM, DNA-PKcs plays a key role in DNA repair in HR and NHEJ; the role of DNA-PKcs in DNA repair has been reviewed comprehensively by Goodwin et al.^50^ In addition to these well documented DNA damage repair pathways, DNA-PKcs is also involved with various processes that are thought to be important for tumor progression, such as cell cycle progression, transcription, and telomere maintenance,^51^ making DNA-PKcs an attractive target for cancer therapy.

DNA-PKcs is dysregulated in numerous cancers such as melanoma, where it encourages angiogenesis and tumor migration, and it has been discovered that the DNA-PKcs is associated with the secretion of prometastatic proteins through modification of the tumor microenvironment.^52^ DNA-PKcs dysregulation has also been observed in hepatocellular carcinoma^53^ and myeloma^54^ and is associated with radioresistance in cancers including thyroid,^55^ oral cavity,^56^ and cervical cancer.^57^

ATR is not directly associated with the DNA damage response; however it is involved with protecting cells from replication stress.^58^ It does this through preventing the collapse of the replication fork and inducing G2/M arrest by activating the checkpoint kinase CHK1. Cancer cells rapidly proliferate and therefore tend to undergo much replication stress and thus are particularly dependent on ATR. ATR has been observed to show SL with ATM/CHK2/p53: due to the amount of DSBs that are formed when ATR is inhibited, inhibiting key players in the DSB pathway is likely to result in an SL relationship. Of these SL relationships, ATR-ATM seems to be the most promising, as ATM is involved in both DNA repair in addition to cell cycle checkpoint activation. Furthermore, a deficiency in ATR has been shown to be synthetically lethal with agents that cause DNA stress, such as nonspecific chemotherapeutics. These SL relationships have been seen in various cancer cells including leukemia,^59^ pancreatic,^60^ and gastric cancer;^61^ in some cases, the SL phenotype was only observed in combination with DNA damaging agents.

ATR has also been shown to be synthetically lethal with CHK1, where small molecule-induced synthetic lethality has been reported, resulting in the selective killing of cancer cells.^62^ It has been hypothesized that the mechanism for SL of these two targets is that CHK1 inhibition increases replication stress through the deregulation of origin firing.^63^ The result of this is the stalling of DNA replication forks, increasing the concentration of ssDNA that must be repaired by the ATR-dependent replication protein A; however, due to ATR inhibition, this cannot occur and therefore a large amount of DSB accumulates, thus causing cell death.

**Figure 3:**
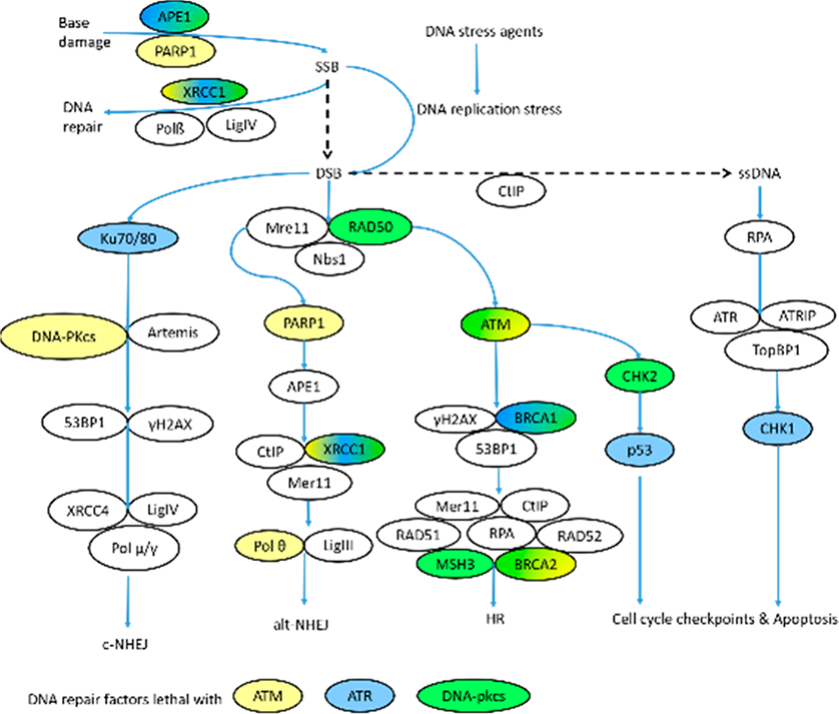
Demonstration of ATM, ATR, and DNA-PKcs involvement in DNA repair networks. Adapted from Trends in Cancer, Vol. 4, Issue (11), , Omar L. Kantidze, Artem K. Velichko, Artem V. Luzhin, Nadezhda V. Petrova, Sergey V. Razin, Synthetically Lethal Interactions of ATM, ATR, and DNAPKcs10.1016/j.trecan.2018.09.00730352678,^42^ Page 762, Copyright (2018) with permission from Elsevier.

ATR, ATM, and DNA-PKcs are promising targets for impeding DNA damage repair, as all are involved in the disruption of the cell cycle and the initiation of the DNA damage repair process (Figure 3). Currently multiple ATR inhibitors are in clinical trials, the data for which can be seen in Table 1.

**Table 1:** List of Current Clinical Trials Involving Inhibition of ATR

trial identifier	drug	phase	summary	status and accession date
NCT04266912	**6**	I/II	A combination study of avelumab with **6**/nediseritib in participants with metastatic or inoperable tumors with deficient DNA damage repair.	Recruiting
	Avelumab (PD-L1 antibody)			February 12, 2020
	Nedisertib (DNA-PKi)			
NCT02589522	**6**	I	Studying the side effects and dose of **6** in combination with whole brain radiation in participants with non-small-cell lung cancer, small cell lung cancer, or neuroendocrine tumors that have spread to the brain.	Recruiting
				October 28, 2015
NCT02630199	**11**	I	A combination study assessing the safety and tolerability of **6** and paciltaxel in participating metastatic cancer patients who have failed standard chemotherapy.	Recruiting
	Paclitaxel (nonspecific chemotherapeutic)			December 15, 2015
NCT03896503	**6**	II	Combination study comparing **6** and topotecan HCl to topotecan HCl as a single agent in participants with relapsed/extrapulmonary small cell lung cancer.	Recruiting
	Topotecan HCl (nonspecific chemotherapeutic)			April 1, 2019
NCT04149145	**3**	I	A combination study of **3** and **6** in participants with PARP-resistant recurrent ovarian cancer, to assess the safety of the combination, the response rate, and the percentage of participants who proceed to 6 months progression-free survival among patients who have developed PARPi resistance and to assess the indicators of response and progression of this disease following the combination therapy.	Not yet recruiting
	**6**			November 4, 2019
NCT03527147	AZD9150	I	A study to investigate various targeted agents’ safety, tolerability, efficacy, and PK/PD profile in participants with relapsed or refractory aggressive non-Hodgkin’s lymphoma.	Recruiting
	Acalabrutinib			May 17, 2018
	**11**			
	Hu5F9-G4			
	Rituximab			
	AZD5153			
NCT03462342	**1**	II	A combination study to assess the safety, tolerability, response rate, and progression-free survival of **1** and **11** in women with recurrent ovarian cancer (platinum-sensitive or platinum-resistant).	Recruiting
	**11**			March 12, 2018
NCT04065269	**1**	II	A combination trial of **1** and **11** in participants with gynecological cancers with ARId1A loss or no loss, to assess response rates in groups of participants selected based on their cancer cell subtype and the presence of an abnormality in ARID1A gene.	Recruiting
	**11**			August 22, 2019
NCT02723864	Veliparib	I	A combination study to assess the safety, tolerability, and maximum dose of **6** and veliparib combined with cisplatin in participants with advanced refractory solid tumors.	Recruiting
	**6**			March 31, 2016
NCT04267939	**3**	I	A combination study to assess the safety, tolerability, and maximum/recommended phase II dose of **8** and **3** in participants with recurrent advanced solid tumors and ovarian cancer.	Recruiting
	**8**			February 13, 2020
NCT03188965	**8**	I	A study to assess the safety, tolerability, maximum dose, and response rate of **8** in participants with advanced solid tumors and lymphomas.	Recruiting
				July 16, 2017
NCT04095273	**8**	I	A combination study to assess the safety/tolerability, PD/PK profile, and optimum dose for **8** and pembrolizumab in participants with advanced solid tumors.	Recruiting
	Pembrolizumab (PD-1 antibody)			September 19, 2019
NCT03641547	**6**	I	A combination study of **6** with standard of care chemotherapeutics to establish the safety, tolerability, maximum tolerated dose, and efficacy in participants with esophageal and other cancers.	Recruiting
	Cisplatin			August 22, 2018
	Capecitabine			
	Radiotherapy (nonspecific chemotherapeutics)			
NCT02223923	**11**	I	A study to assess **11** in combination with radiotherapy. The study will investigate **11**’s safety, tolerability, dose, and dosing schedule while assessing preliminary drug response rates in participants with solid tumors.	Active, not recruiting
	Radiotherapy			August 22, 2014
NCT03682289	**1**	II	A study to assess **11** as a monotherapy and in a separate arm of the trial in combination with **1**, in participants with metastatic renal cell carcinoma, urothelial carcinoma, all pancreatic cancers, or other solid tumors. This study will assess objective response rate, median duration of response, median progression-free survival, and progression-free survival rate at 6 and 12 months. It will also further assess **11**’s safety.	Recruiting,
	**11**			September 24, 2018
NCT03641313	**6**	II	A combination study to determine the overall response rate of **6** and irinotecan in participants with progressive, metastatic, or unresectable TP53 mutant gastric or gastroesophageal junction cancer. Furthermore, it seeks to assess the duration of response, time to progression, progression-free survival, and OS in participants with the combination of drugs in comparison to irinotecan alone and in participants with other DNA damage repair defects such as mutations in RCA1, BRCA2, MRE11, RAD50, RAD51, RAD52, RAD54L, NBN, ATM, H2AX, PALB2, RPA, BRIP1, BARD1, ATR, ATRX, CHK1, CHK2, MDM2, MDM4, FANCA, FANCC, FANCD2, FANCE, FANCF, FANCG and FANCL. Lastly, it seeks to compare the combination of **6** and irinotecan in participants who are Pt-sensitive and resistant.	Not yet recruiting
	Irinotecan (topoisomerase inhibitor)			August 22, 2018
NCT02627443	**6**	I/II	A combination study to assess the safety, tolerability, maximum tolerated dose of **6**, carboplatin, and gemcitabine HCl in patients with recurrent and metastatic ovarian, primary peritoneal, or fallopian tube cancer. Furthermore, this study seeks to assess the maximum tolerated dose effect on overall survival, duration of response, and progression-free survival.	Recruiting
	Carboplatin (nonspecific chemotherapeutic)			December 11, 2015
	Gemcitabine HCl (Cytotoxic chemotherapeutic)			
NCT02567409	**6**	II	A combination study to assess whether **6** in combination with standard of care therapeutics improves progression-free survival, in comparison to standard of chemotherapy as a monotherapy in patients with metastatic urothelial cancer. Furthermore, this study seeks to compare overall survival, tumor response rate, and safety between the two study arms. Lastly, to assess the role of p53 in predicting response to **6**.	Active, not recruiting
	Cisplatin (nonspecific chemotherapeutic)			October 5, 2015
	Gemcitabine HCl (cytotoxic chemotherapeutic)			
NCT02487095	**6**	I/II	A study to assess the safety and efficacy of **6** and topotecan in treating small cell lung cancer.	Recruiting
	Topotecan (nonspecific chemotherapeutic)			July 1, 2015
NCT03787680	**1**	II	A combination study is to test the effectiveness, safety, and tolerability of **1** and **11** for all participants with metastatic castration-resistant prostate cancer.	Recruiting
	**11**			December 26, 2018
NCT02595892	**6**	II	A combination study to assess the progression-free survival of **6** and gemcitabine in comparison to gemcitabine as a monotherapy in participants with recurrent ovarian, primary peritoneal, or fallopian tube cancer. Across both arms will also be tested overall response rate, safety profiles, progression-free survival at 6 months, clinical benefit rate, duration of response, cancer antigen (CA)125 reduction, and overall survival.	Active, not recruiting
	Gemcitabine (cytotoxic chemotherapeutic)			November 4, 2015
NCT03669601	**11**	I	A dose escalation trial to assess the safety of **11** in combination with gemcitabine in participants with advanced solid tumors.	Recruiting
	Gemcitabine (cytotoxic chemotherapeutic)			September 13, 2018
NCT03328273	**11**	I/II	A combination study that evaluates the safety, pharmacokinetics, pharmacodynamics, and efficacy of acalabrutinib and **11**.	Recruiting
	Acalabrutinib (BTK inhibitor)			November 1, 2017
NCT04052555	**6**	I	A combination study to test the recommended dose for phase II trials of **6** in combination with standard of care radiotherapy in participants with triple-negative or estrogen receptor and/or progesterone receptor positive, HER-2 negative breast cancer.	Recruiting
	Radiotherapy			August 12, 2019
NCT02567422	**6**	I	A combination study to assess the safety, tolerability, and maximum tolerated dose of **6** in combination with standard of care cisplatin and radiotherapy in participants with locally advanced head and neck squamous cell carcinoma.	Recruiting
	Cisplatin (nonspecific chemotherapeutic)			October 5, 2015
	Radiotherapy			
NCT02595931	**6**	I	A study to assess the safety, tolerability, and maximum tolerated dose of **6** and irinotecan hydrochloride in treating participants with metastatic or unresectable DNA damage repair deficient solid tumors.	Recruiting
	Irinotecan HCl (topoisomerase inhibitor)			November 4, 2015
NCT04266912	**6**	I/II	A study to assess the safety, tolerability, and maximum tolerated dose of **6** and avelumab in treating participants with metastatic or unresectable DNA damage repair deficient solid tumors.	Recruiting
	Avelumab (PD-L1 antibody)			February 12, 2020
NCT03517969	**6**	II	A combination study that studies response rate of **20** and carboplatin with and without docetaxal in participants with castration-resistant prostate cancer.	Recruiting
	Carboplatin (nonspecific chemotherapeutic)			May 8, 2018
	Docetaxel (microtubules binder)			

**Figure 4:**
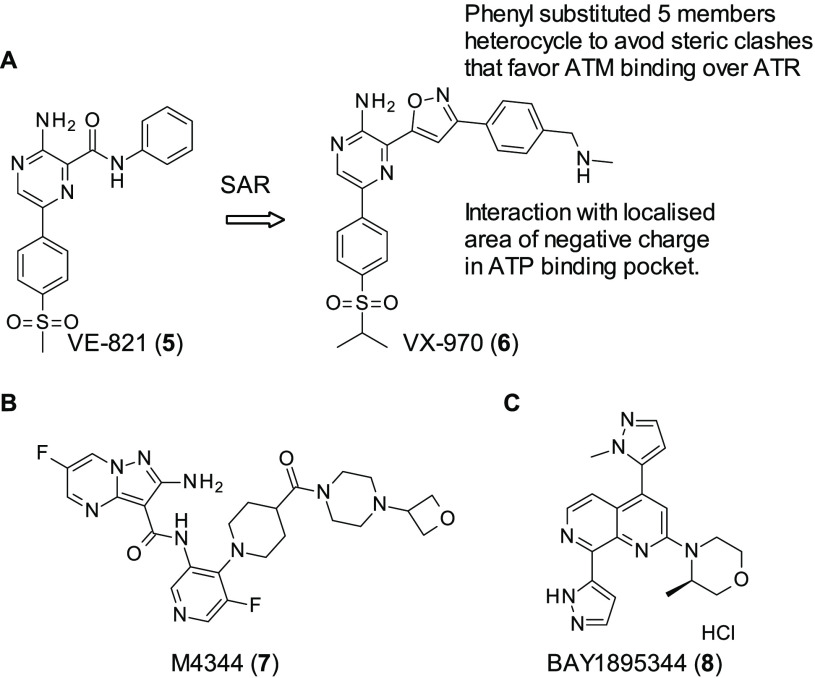
(A) SAR study around ATR inhibitor VE-821 (**5**) to get VX-970 (**6**). (B) Structure of ATR inhibitor M4344 (**7**). (C) Structure of BAY1895344 (**8**) identified by Bayer AG as a selective ATR inhibitor.

VE-821 (**5**, Figure 4A) (ATR IC_50_ = 26 nM) is an ATR inhibitor developed by Vertex pharmaceuticals in 2011. This compound is typical of numerous inhibitors from this development program, all of which contain a 2-aminopyrazine binding motif.^64^ While this compound was a useful tool in understanding the underlying biology associated with ATR inhibition, it did not exhibit good solubility, and its metabolic profile, specifically producing aniline-like metabolites, was undesirable for a clinical candidate; thus Vertex began optimization of **5**.^65^ Initial optimization of **5** involved the replacement of the anilide group with various fused bi-cycles: while these were well-tolerated in terms of ATR inhibition, they also demonstrated a loss in selectivity for ATR against ATM. Through *in silico* modeling it was theorized that this loss in selectivity was caused by the bulky fused bi-cycles and the gatekeeper residue of the PIKK tyrosine kinase family, causing rotation of the bound inhibitor. This in turn led to a further steric clash between Pro2755 of ATM and the isopropylsulfone group of the inhibitor that caused tighter ATM binding. With this information in hand, Vertex theorized five-membered phenyl substituted heterocycles would avoid these steric clashes and therefore would be able to maintain selectivity, as they more closely resemble the anilide moiety present in **5**. Additionally, with substitution upon the phenyl ring, a localized highly negatively charged area of the ATP binding pocket could be exploited to further increase potency, selectivity, and solubility. These SAR studies resulted in the eventual synthesis of VX-970 (**6**, Figure 4A), which shows picomolar ATR activity (IC_50_ = 0.17 nM) and >250 degree of selectivity over ATM. In cells, it was able to sensitize colorectal cancer cells to cisplatin, using less than 50 nM **6**. Furthermore, it demonstrated a good pharmacokinetic profile (Cl = 26 mL min^–1^ kg^–1^; *V*_ss_ = 21 L/kg; *T*_1/2_ = 11.6 h), in addition to good bioavailability, making it ideal for further *in vivo* studies. **6** is a first-in-class inhibitor and has been tested both as a monotherapy and with nonspecific chemotherapeutics such as topotecan, carboplatin, gemcitabine, and cisplatin.^66−69^**6** presented promising results as a monotherapy, showing a complete response in one patient for greater than 19 months, with no associated toxicity. However, in phase I trials with the nonspecific chemotherapeutics, bone marrow toxicity was observed. The toxicity issues observed are somewhat common for nonspecific chemotherapeutics, and in this instance the combinatorial approach yielded no benefit over the monotherapeutic approach. Several trials are still ongoing for **6** in numerous cancer subtypes. **6** has been acquired by Merck KGaA under the new name M6620; additionally Merck has entered a second ATR inhibitor using a different scaffold into clinical trials, M4344 (**7**, Figure 4B) (ATR *K*_i_ ≤ 150 pM).^70^ The development of **7** has yet to be published, with preclinical data only being presented at a congress.

BAY1895344 (**8**, Figure 4C) was developed by Bayer AG as described in a patent (**8** is example 111 in patent WO 2016/020320),^71^ through a high-throughput screen HTS. **8** is a novel potent (7 nM) and selective ATR inhibitor (hitting 6 of 456 kinases tested in the kinome screen). Furthermore, **8** was able to selectively inhibit ATR mediated DNA repair and inhibited proliferation in a range of cancer cell lines, being most active in lymphoma cell lines showing an IC_50_ of 9 nM.^72^ It exhibits strong monotherapy efficacy in cancers with impeded DNA damage repair. In addition to this, it synergizes well with nonspecific chemotherapeutics (cisplatin and carboplatin) and other DNA damaging agents (external beam radiotherapy).

It is currently under investigation in clinical trials in patients with advanced solid tumors and lymphomas (Table 1). Lastly, **8** showed synergism with **1** in BRCA1/2 deficient breast cancer cells, suggesting a synthetically lethal interaction; these results were mirrored *in vivo* in breast cancer models.

**Figure 5:**
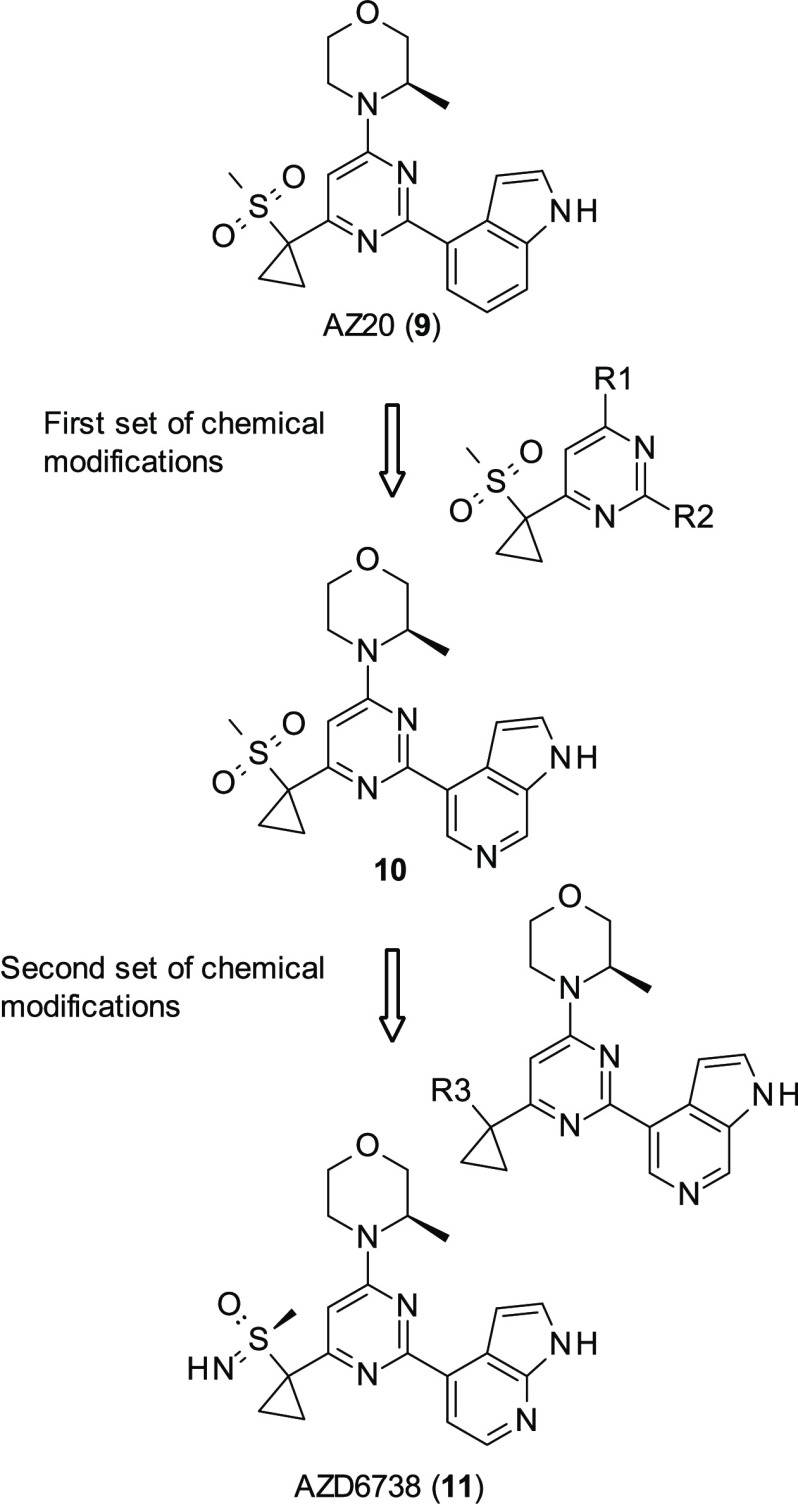
Chemical modifications introduced in AZ20 (**9**) to improve solubility and reduce CYP3A4 activity.

AstraZeneca (AZ) had previously developed a series of potent and selective ATR inhibitors, of which the best performing was AZ20 (**9**, Figure 5) (ATR IC_50_ = 5 nM).^73^ However, while this compound was able to prevent the growth of ATM-deficient xenograph models in tolerated dosages, its aqueous solubility was poor. Furthermore, it was found to hit cytochrome P450 3A4 (CYP3A4), thereby increasing the chance of drug–drug interaction: given the unlikeliness of ATR inhibitors being administered as a monotherapy, this would stop it progressing through further studies. Therefore, a drug-discovery program was initiated, looking to keep **9**’s activity but with improved aqueous solubility and lessened CYP3A4 activity.^74^ Initial SAR looked at making modifications at the R1 and R2 positions (Figure 5); alterations at the R1 position did not reduce CYP3A4 activity and therefore were quickly abandoned. Modifications at the R2 position began with simple ring substitutions; while this did reduce CYP3A4 activity, it also decreased ATR activity, suggesting that the indole group was the reason for CYP3A4 activity. Ring switch to 6- or 7-azaindole and to 2-aminobenzimidazole showed similar ATR activity in cell-based and biochemical assays to AZ20 while reducing CYP3A4 activity. The best of the series was **10** (Figure 5) (ATR IC_50_ = 5 nM). Exploration of substitution of the benzimidazole was attempted; however, none of the changes at this position proved fruitful as all modifications in this position resulted in either loss in ATR inhibition or physicochemical properties, such as aqueous solubility.

Subsequent modifications began at the sulfone group, looking to expand upon **10**. While initial replacement of the sulfone with sulfoxide resulted in promising molecules, the metabolic risks associated with sulfoxides were deemed too high, and therefore AZ did not pursue this avenue of research. However, substitution with various sulfoximines resulted in AZD6738 (**11**, Figure 5) (ATR IC_50_ = 4 nM), which achieved all goals of the lead-development program, with decreased lipophillicity and improved aqueous solubility, in addition to reduced CYP3A4 activity in comparison to **9**.

Compound **11** was taken through to further screening for its biological, physiochemical, and ADME properties. It was found to be the lead compound from this series due to its excellent solubility, permeability, and selectivity (only hitting ∼60% for PIK3C2G and CLK4 out of the 409 kinases tested in the kinome screen) and demonstrated profound antitumor activity. **11** has progressed in multiple phase I/II trials, and while some are in combination with nonspecific chemotherapeutics such as carboplatin (NCT02264678), it has also entered trials with the PARP inhibitor **1**, suggesting synthetic lethality (NCT03462342, NCT03330847), with further preclinical studies underway for other combinations with **1**.^75^ Overall, the clinical candidate **11** has a favorable pharmacokinetic profile for once or twice daily dosing, achieving biological efficacy at moderate dosages. The selectivity of this compound should be noted as it makes it an ideal tool for further exploration of ATR’s synthetically lethal partners such as ATM and CHK1.

**Figure 6:**
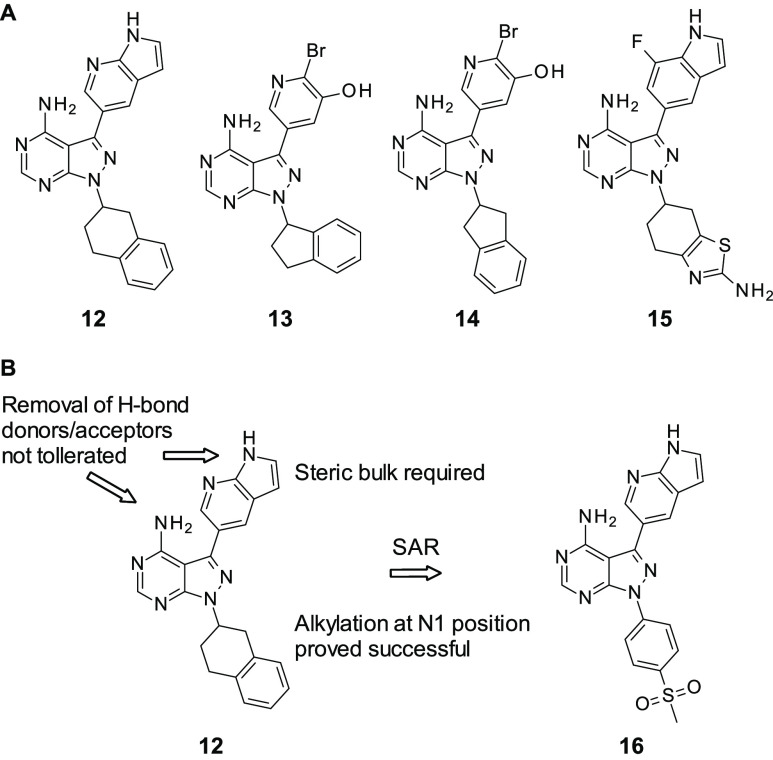
(A) Hit compounds from Ramachandran et al. (B) Optimization of **12** to **16**.

It has been discovered that homology exists between PI3K and ATR;^76^ therefore, it was hoped that it could be harnessed for the discovery of novel ATR inhibitors. Ramachandran et al. had previously been working on dual inhibitors of Bruton’s tyrosine kinase (BTK) and PI3K:^77^ this was used as a starting point for the design of ATR inhibitors.^78^ From the 299 selected compounds in the BTK series, 11 showed inhibition of >50% at 5 μM while only 1 showed inhibition of >50% at 500 nM. Interestingly, despite the homology between the two kinases, there was poor overlap between the inhibition of both, suggesting that the scaffold utilized in this series is differentiated somewhat from other ATR inhibitors (**8**, **9**–**11**), which were also developed from PI3K inhibitors. Of the originally tested compounds, **12** (Figure 6A) stood out as the prime target for further optimization not only because of it being the strongest inhibitor of ATR from this series (IC_50_ = 0.220 μM) but also because it showed relatively low activity against BRK and PI3K (BRK inh = 24%, PI3Kd inh = 52% at 100 nM). **12** was tested as a racemic mixture as there was less than a 2-fold difference in ATR activity between the two enantiomers (IC_50_ = 0.304 μM vs 0.158 μM). This was tested with three other compounds (**13**–**15**, Figure 6) that also showed good activity (in the low micromolar range). All four of these compounds share the same common 4-aminopyrazolopyrimidine scaffold functionalized at N1 with a partially aromatic bicyclic system: it was discovered that removal of the hydrogen bond donors/acceptors present in this core scaffold was not well-tolerated, causing either a partial or complete loss of activity against ATR. In addition to this, replacement of the azaindolyl group with an aminopyridine group which displayed the same hydrogen bond acceptors/donors was not tolerated, suggesting the steric bulk present in the aminopyrazolopyrimidine group is necessary for ATR inhibition. Follow-up SAR was conducted, introducing new bulky groups at the C3 position, and yet none of these were able to improve on the activity of **12**. Therefore the focus of the SAR studies turned to alkylation at the N1 position. While the majority of modifications at this position did not yield a significant improvement on the activity of **12**, compound **16** (Figure 6B), which takes influence from Vertex’s **5**(65) featuring a phenylsulfonyl group, managed to yield the most potent ATR inhibitor in this series to date with a 3-fold improvement in activity (IC_50_ = 66 nM) over **12**.

**16** was compared to the clinical candidate ATR inhibitors **6** (IC_50_ = 15 nM) and **11** (IC_50_ = 86 nM) and in the same testing conditions showed similar levels of ATR inhibition. The lipophilic efficiency of **16** (5.8) and oxidative stability were in the same region of the two literature compounds. **16** was also tested for selectivity in a kinome screen of 394 kinases: of these, 10 were inhibited at greater than 70% at 1 μM and 76 were inhibited at greater than 70% at the 10 μM, with only breast tumor kinase (BTK) being inhibited to a greater degree than ATR. Lastly, **16** was tested *in vivo* in mice, where it showed good clearance but poor bioavailability, suggesting further optimization is required. Given ATR’s potential for small molecule-induced synthetic lethality, any optimization of this compound should be monitored with interest.

**Figure 7:**
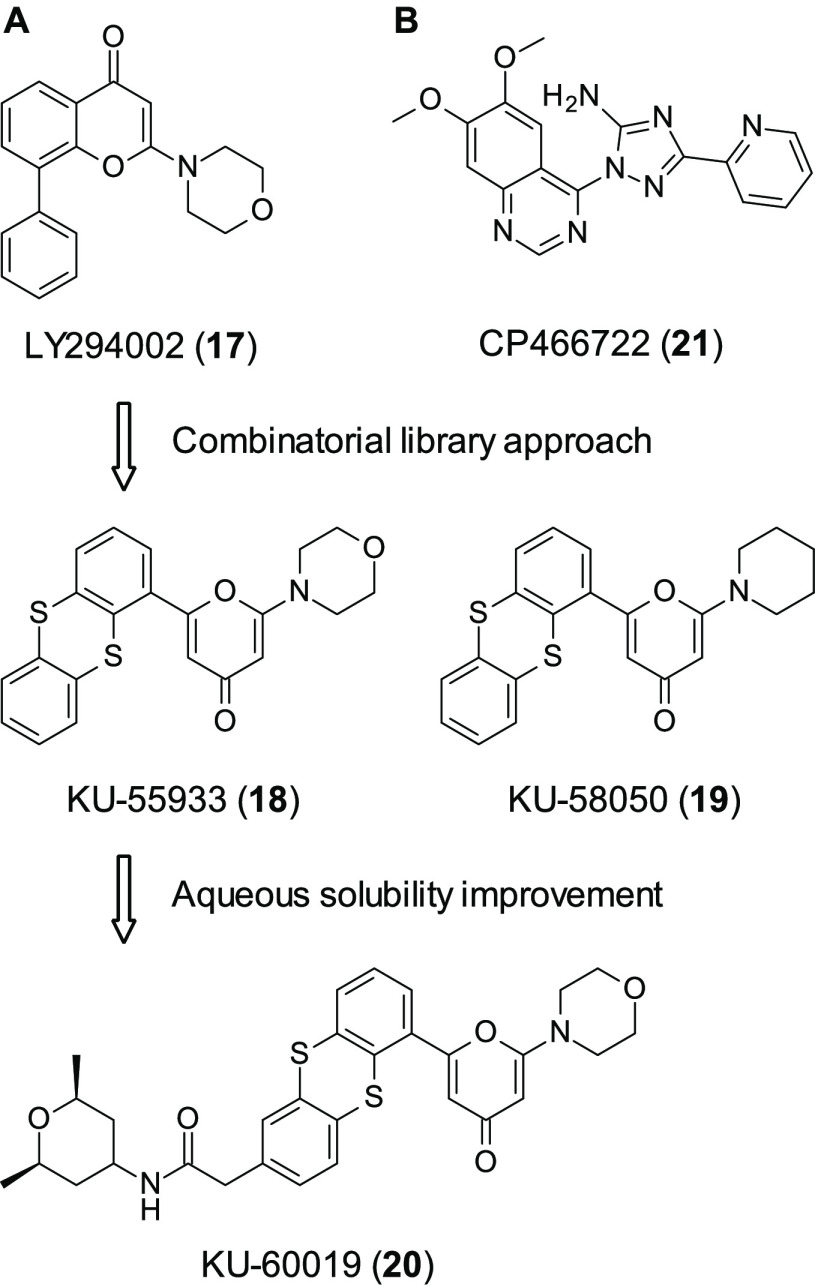
(A) Development of ATM inhibitor LY294002 (**17**) into KU-60019 (**20**). (B) Structure of ATM inhibitor CP466722 (**21**).

In 2004, Hickson et al. developed the first selective ATM inhibitors, as up to this date all molecules that had inhibited ATM were nonspecific PIKK and PI3k inhibitors such as caffeine. Through a combinatorial library approach based around the nonspecific PIKK and PI3k inhibitor LY294002 (**17**, Figure 7A), the group developed the ATP competitive inhibitor KU-55933 (**18**, Figure 7A), which demonstrated in biochemical assays an IC_50_ of 12.9 ± 0.1 nM, with at least a 100-fold degree of selectivity over 60 other selected similar kinases.^79^ In these studies, interesting structural information was gleaned when replacing the oxygen in the morpholine ring (Figure 7). This resulted in a significant drop in potency in KU-58050 (**19**, Figure 7A) (2.96 ± 0.44 μM), a 200-fold drop in potency in comparison to **18**, showing the importance of the oxygen at this position. Furthermore, **18** was able to sensitize cells to ionizing radiation and nonspecific chemotherapeutics, thus increasing the amount of DSB.

Following on from this work, in 2009 Golding et al. developed KU-60019 (**20**, Figure 7A) by expanding upon the thianthrene group with the aim of improving **18**’s bioavailability and pharmacokinetics.^80^**20** was more water-soluble, was able to double the potency with respect to **18** (6.3 nM), and was more selective, showing a 240- and 1600-fold degree of selectivity for DNA-PKcs and ATR, respectively. **20** is better at sensitizing cells to ionizing radiation, showing an enhancement of 4.4 compared to 1.6 from **18**, when both compounds were administered to glioma cells at 10 μM. In scratch assays, **20** was able to reduce invasion *in vitro* in U87 cells and a greater than 70% drop in invasion was observed in a dose-dependent manner. In the more invasive U1242 glioma cells, a greater than 50% decrease in invasion was observed. It is thought that **20** showed this anti-invasive phenotype by acting on AKT and MEK/ERK prosurvival pathways. **20** is currently undergoing a clinical trial (Table 2) in combination studies with CHK2 inhibitors; should this work, it could indicate small molecule-induced synthetic lethality.

**Table 2:** Current Clinical Trials Involving ATM Inhibitors

trial identifer	drug	phase	summary	status and accession date
NCT03423628	**29**	I	A study to assess the safety, tolerability, and PK of increasing doses of **29** in combination with distinct regimens of radiation therapy in participants with brain cancer.	Recruiting
	Radiotherapy			February 6, 2018
NCT03571438	CX4945 (CK2i)	Not applicable	A combination study to compare CX4945 and **20** to standard of care treatments directly on organotypic cultures of tumors from participants, measuring incidents of cell death.	Recruiting
	**20**			June 27, 2018
	Sunitinib (multiple RTKi)			
	Pazopanib (multiple RTKi)			
	Temsirolimus (mTORi)			
NCT02588105	**1**	I	A study to test the safety, tolerance PK/PD, and initial efficacy of **28** as a monotherapy and in combination with the other listed drugs.	Active, not recruiting October 27, 2015
	**28**			
	Irinotecan (topoisomerase inhibitor)			
	Fluorouracil (thymidylate synthase inhibitor)			
	Folinic acid			

In 2008 Rainey et al., through use of an *in vitro* kinase screen of approximately 1500 small molecules, identified CP466722 (**21**, Figure 7B) (ATM IC_50_ = 12.9 nM).^81^ This was able to inhibit ATM activity *in vitro* but did not show activity against the closely related kinases in the PI3k family. **21** showed a lack of toxicity and the ability to inhibit ATM in human and mouse cells, the latter being important as the group wished to test the compounds *in vivo* in murine models. Furthermore, **21** was able to sensitize cancer cells to ionizing radiation. It was also demonstrated that **21** was a reversible binder, shown to bind quickly and effectively for at least 8 h in tissue culture; however, upon 30 min of wash off, ATM-dependent phosphorylation events were restored. This reversibility is key due to the important role ATM plays in the cell cycle.^82^ This compound has yet to be used for further studies.

**Figure 8:**
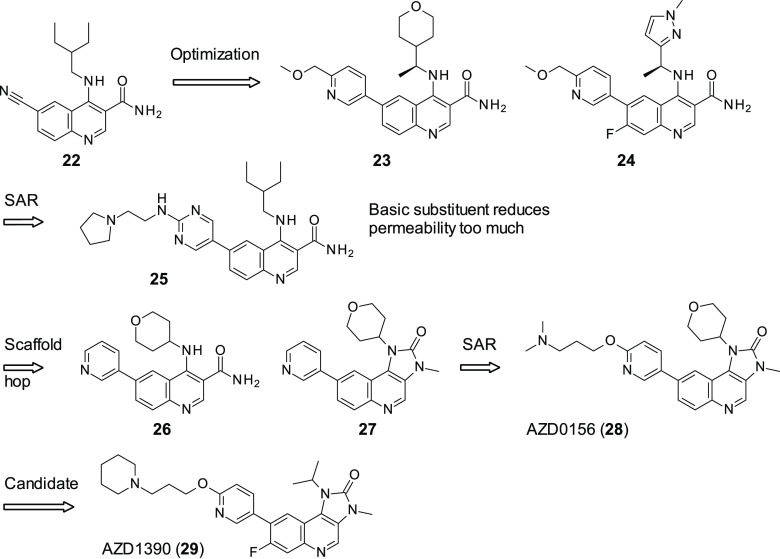
Development by AstraZeneca of ATM inhibitor from hit **22** into candidate AZD1390 (**29**) through the lead AZD156 (**28**).

AstraZeneca has acquired KuDOS pharmaceuticals and has continued building upon the progress made by **20**. Through optimization of an initial screening hit, **22** (Figure 8) (ATR cell IC_50_ = 82 nM), AZ was able to develop two further compounds: **23** (Figure 8) (ATR cell IC_50_ = 46 nM) and **24** (Figure 8) (ATR cell IC_50_ = 33 nM). **23** and **24** allowed exploration of the ATM inhibitors *in vivo*.^83^ At an oral dosage of 100 mg/kg QD, **23** was able to increase sensitivity to ionizing radiation in a HT29 xenograph mouse model.^84^ Both **23** and **24** were able to enhance the efficacy of irinotecan in a SW620 mouse xenograft models, with **24** able to contribute to tumor regression.^83,84^ In line with other ATM inhibitors, **23** and **24** were ineffective without a DNA damaging agent to induce DSB. **24** showed a favorable reduction in activity against hERG relative to **23** (IC_50_ of 22 and 4.5 μM, respectively) and therefore had a lower chance of cardiovascular issues later on in clinical development, suggesting that **24** would be the better clinical candidate going forward.^85^ Through pharmacological modeling, it was found that **24** would have a low half-life (only 4 h) in humans, and therefore the predicted dose would be 700 mg QD, with a maximum unbound concentration *C*_max_ of 1.3 μM. In addition, other calculated parameters such as predicted clinically efficacious dose and maximum absorbable dose (*D*_abs_) were unfavorable; therefore AZ concluded that the probable chance of attrition was too high. A further development program was initiated with an aim to produce molecules with a reduced predicted clinical dose and an increased *D*_abs_. AZ set about attempting to increase their compounds’ volume of distribution (*V*_ss_), as pharmacokinetic half-life is calculated through *V*_ss_ and clearance (CL). As **24** already showed a low metabolic turnover, further reduction of CL would be challenging; therefore, *V*_ss_ was chosen as the parameter to optimize. This began with the attempted inclusion of a basic substituent with the molecule, as it has been shown previously that basic compounds increase *V*_ss_ with respect to acidic or neutral compounds,^86^ with the idea of keeping other parameters the same. While **25** (ATR cell IC_50_ = 8.6 nM) showed an increase in ATM inhibition with respect to **24**, a drop in permeability to unacceptable levels occurred, meaning that basic substituents would be unlikely to be tolerated going forward. In an effort to increase permeability by reducing the number of hydrogen bond donors, a scaffold hop was attempted. This was done using a pseudo-ring system and a tri-cycle system, imidazo[4,5-*c*]quinolin-2-one, which was selected from previous AZ in-house data. This scaffold has also seen success in ATM inhibition as an off-target in the dual PI3K/mTOR inhibitor, NVP-BEZ235, which has entered clinical trials.^87,88^ Of these compounds, **26** and **27** (Figure 8) were the most potent (ATM IC_50_ = 0.95 and 0.36 μM, respectively), with **27** showing reduced aqueous solubility. Given the promising results with this scaffold, motifs from **23** and **24** were modeled upon the imidazo[4,5-*c*]quinolin-2-one scaffold to create a second series. Through in-depth SAR of the imidazo[4,5-*c*]quinolin-2-one scaffold, AZ eventually identified AZD0156 (**28**, Figure 8) as the lead compound. **28** showed an excellent affinity for ATM (IC_50_ = 0.04 nM in biochemical assays, 0.57 nM in cells) and an exceptional degree of selectivity, with only 2 of the 397 kinases tested in the kinome screening showing greater than 70% inhibition (mTOR, 93%; LRRK2, 87%). **28** showed desirable levels of unbound drug in human plus two other mammalian species (rat and dog) and acceptable permeability, and it did not target any of the main five isoforms of P450.^89^ In addition to this, the predicted pharmacokinetics of **28** were favorable, with low to moderate clearance in man (∼8 mL min^–1^ kg^–1^), moderate to high *V*_ss_ (5.8 L/kg), and high oral bioavailability (66%). In docking studies, **28** showed good complementarity to ATM’s ATP binding site, displaying two interactions with the kinase hinge region (Cys2770 and Lys2717) and one in the back binding pocket (Tyr2755). In addition to this, the basic amine, present in **28**, is predicted to be surrounded by three acidic residues (Asp2725, Asp2720, and Asp288). **28** demonstrated the ability to increase the effectiveness of DSB-inducing agents such as irinotecan. In combination studies, irinotecan (50 mg/kg ip Q7D) and **28** (dosed orally at 20 mg/kg QD) were tolerated, causing tumor regression in SW620 xenograph models in immunocompromised mice. However, as expected, when **28** was administered as a monotherapy, it showed no effect. All these results indicate that **28** is a first-in-class selective ATM inhibitor with promising potential applications for cancer therapy; it is currently involved in a clinical trial highlighted in Table 2.

With the **28** series showing success, further screens were conducted on related hits to try and find other clinical candidates. Given rates of attrition in drug development, having a second, backup molecule in the pipeline provides a level of security. AZD1390 (**29**, Figure 8) was discovered in an *in vitro* screen, with a similar structure as **28**. It was designed to perform well in the following parameters: ATM autophosphorylation activity (IC_50_ = 0.09 nM); selectivity against closely related PIKK family kinases (ATR, DNA-PK, mTOR (all IC_50_ ≥ 1 μM)); general selectivity across the kinome; lack of activity in novel dual-transfected human MDR1 and BCRP efflux transporters assays.^90^ In terms of general selectivity, **29** was tested against a panel of 121 kinases and showed ≥50% inhibition against three targets (CSF1R, NUAK1, and SGK) at 1 μM, with none of the 354 kinases tested showing ≥50% inhibition at 0.1 μM. In a similar fashion to **28**, **29** showed minimal hERG activity (>33.3 and 6.55 μM, respectively). **29** demonstrated good blood–brain barrier (BBB) penetration and has favorable physicochemical and PD/PK properties. Furthermore, it was able to interrupt DNA damage repair in glioblastoma and lung cancer cells. In line with previous studies on ATM inhibitors, **29** was more effective in p53-deficient cells.^91^ It was also demonstrated that treatment of animal orthotropic brain models of glioblastoma and lung cancer brain metastasis (>3 h) with **29** at a concentration above the IC_50,_ in combination with ionizing radiation, blocked tumor growth. It was also observed that a combination of ionizing radiation and **29** caused an increase in apoptosis in comparison to **29** as a monotherapy. In brain cancer models using intracranially implanted xenografts, **29** was tested in combination with ionizing radiation and a triplet combination of **29**, ionizing radiation, and the alkylating agent temozolomide.

While the triplet combination showed more success than the doublet, it appeared to be an additive effect, while ionizing radiation and **29**’s combination were synergistic. In neither study, any behavioral abnormalities were observed because of the administration of **29**, which is key for treatment of the brain. **29** was tested in human-derived glioblastoma models from patients with either temozolomide resistance or sensitivity and either p53 WT or mutant, with the most sensitive to **29** and ionizing radiation as expected being in the p53 mutant models, thus agreeing with their previous data. On the basis of these data, **29** is being considered as a clinical candidate and entered a phase I clinical trial in patients with brain cancer, the information of which are reported in Table 2. These data, in particular the work in glioblastoma, are of great importance to the field. Glioblastoma is the most aggressive cancer of the brain, with poor patient prognosis, a seemingly inevitable rate of recursion, and a median survival rate of 12–14 months with current standard of care therapy.^92^**29** shows the first example of potential SL-related treatment of glioblastoma, one of the most urgent unmet medical needs in oncology.

As highlighted earlier in the review, DNA-PKcs is thought to be synthetically lethal with a number of DNA repair modulating targets, specifically ATM. In this section we highlight some of the DNA-PKcs inhibitors currently in clinical and preclinical studies.

**Figure 9:**
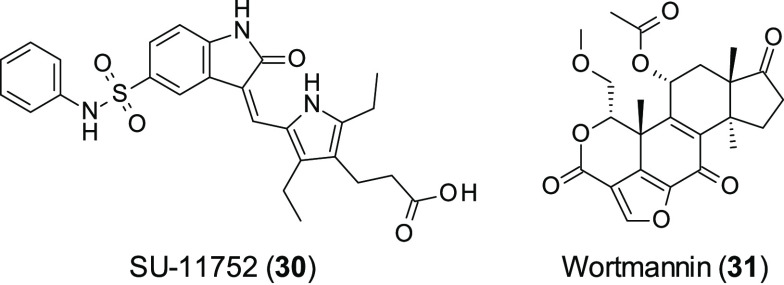
Structure of SU-11752 (**30**) and wortmannin (**31**).

SU11752 (**30**, Figure 9) was the first selective DNA-PK inhibitor to be discovered when it was identified from a library of 3-substituted indolin-2-ones. **30** showed a similar *in vitro* DNA-PK activity to the known PI3K inhibitor wortmanin (**31**, Figure 9) (**30** IC_50_ = 0.13 μM vs **31** IC_50_ = 0.1 μM). The binding mode of **30** was then assessed: **31** has been shown previously to irreversibly bind to DNA-PK, a trait that would be undesirable in a cell cycle modulator;^93^ however **30** was shown to be a reversible ATP competitive inhibitor. Furthermore, **30** was much more selective than **31**, as **30** was 500 times less active against **31**’s primary target PI3Kγ. Additionally **31** inhibits ATM, while in cells **31** was seen to be a poor inhibitor of ATM. **31** was able to sensitize glioblastoma cells to ionizing radiation and disrupt DNA repair.

**Figure 10:**
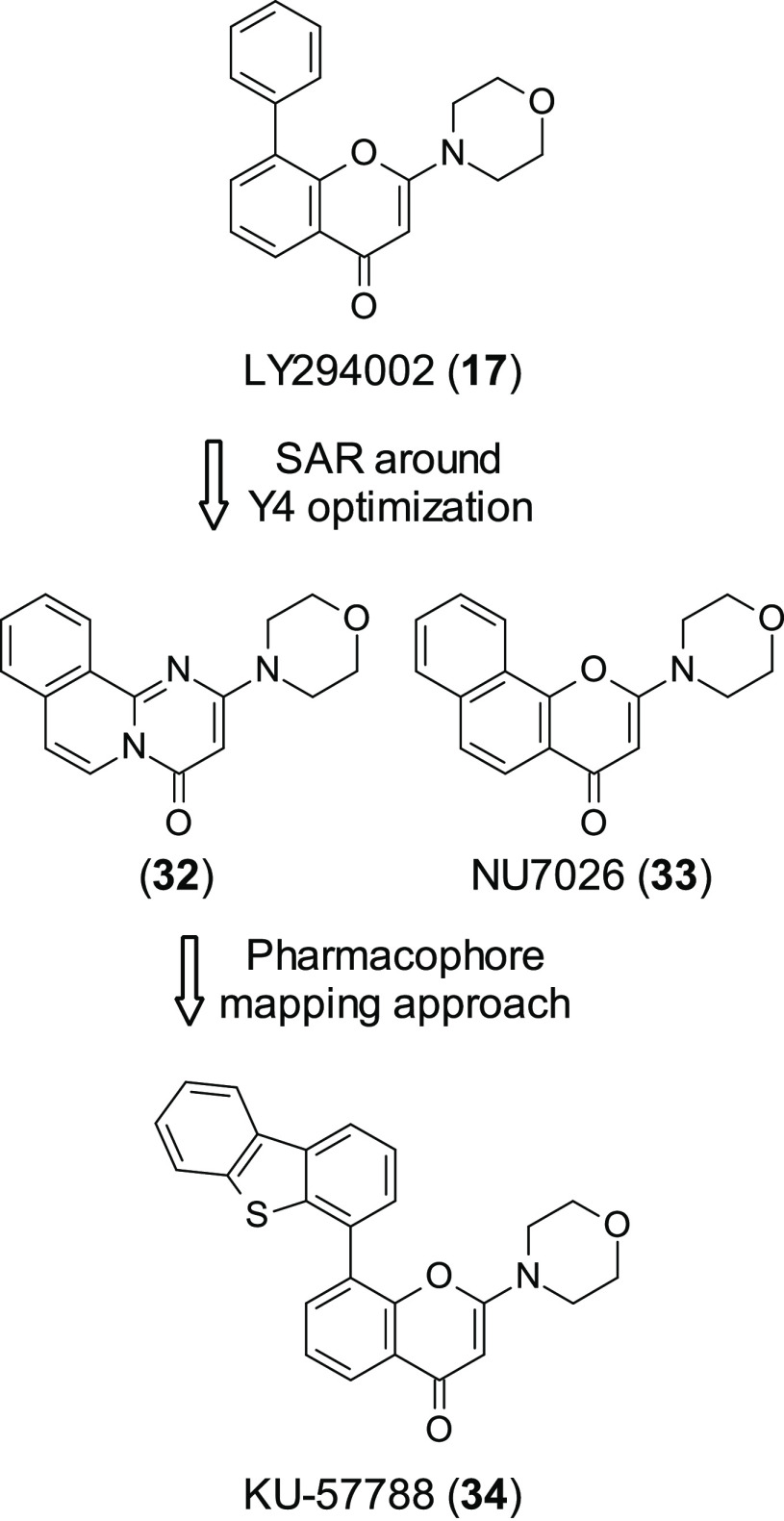
Optimization of PI3K inhibitor **17** resulting in the discovery of KU-5788 (**34**).

**17** is a known PI3K inhibitor (PI3Kα, -β, -δ IC_50_ = 0.5, 0.97, 0.57 μM) that was used as a starting point in the pursuit of novel selective DNA-PK inhibitors in work conducted by Griffin et al. Initial optimization of **17** (Figure 10) resulted in the synthesis of a series of DNA-PK inhibitors, which displayed IC_50_’s in the low micromolar or nanomolar range. The most potent of this series were pyrimidoisoquinolinone (**32**, Figure 10) (DNA-PK IC_50_ = 0.28 μM) and NU7026 (33, Figure 10) (DNA-PK IC_50_ = 0.23 μM), which were able to sensitize tumor cell lines to ionizing radiation and DNA damaging agents.^94^

This work was continued through the use of a pharmacophore mapping approach, replacing or substituting upon the 2-morpholinyl substituent. This approach resulted in the discovery of KU-57788 (**34**, Figure 10) (formerly NU7441).^95^**34** was shown to be a potent DNA-PK inhibitor (IC_50_ = 13 nM) with good selectivity against ATM and ATR (no activity at 100 μM); however it also hit mTOR and PI3K (IC_50_ = 1.7 and 5 nM, respectively). In a similar manner to other DNA-PK inhibitors **34** was able to enhance radiosensitization and the cytotoxicity of etoposide, in Hela cells, while showing no cellular toxicity as a single agent.

**Figure 11:**
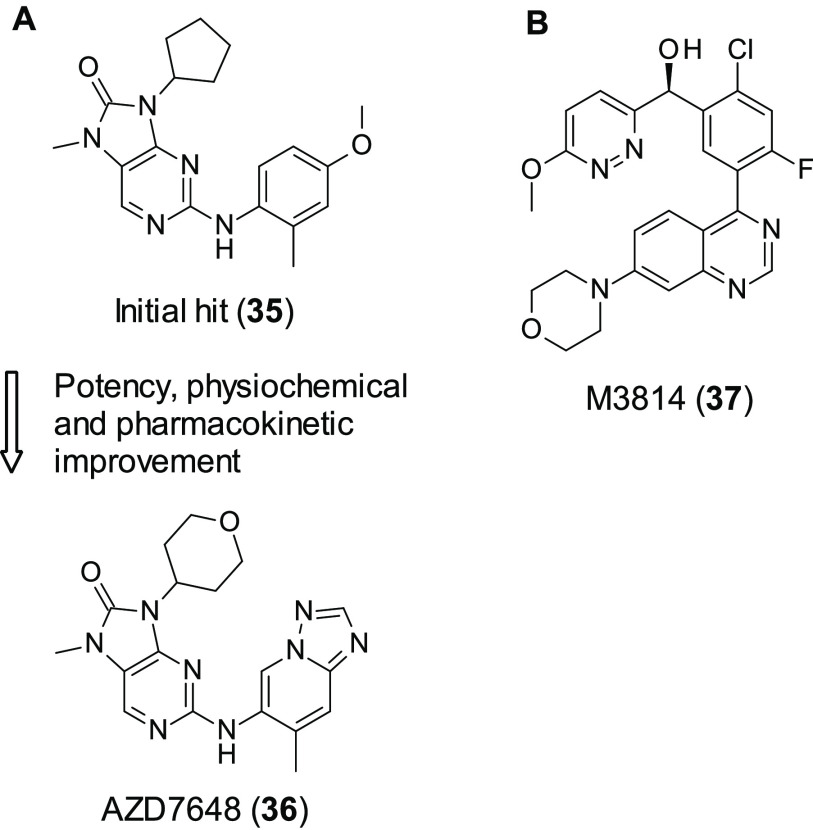
(A) Optimization of DNA-PK inhibitor **35** to AZD7648 (**36**) by AZ. (B) Structure of DNA-PK inhibitor M3814 (**37**) developed by Merck.

AZ set out to discover a selective DNA-PK inhibitor. AZ had observed that many of the current DNA-PK inhibitors showed poor selectivity over related PI3Ks and PIKK family members such as ATM and mTOR. Through use of an in-house screen searching for DNA-PK activity over PI3K, the initial hit (**35**, Figure 11A) (biochemical data not reported) was discovered. **35** was optimized, improving its potency, physiochemical, and pharmacokinetic properties to give AZD7648 (36, Figure 11A).^96^**36** was a potent inhibitor of DNA-PK (IC_50_ = 0.6 nM) and showed good selectivity: in a panel of 397 kinases, only 4 showed inhibition of >50% (PK, PI3Kα, PI3Kδ, and PI3Kγ), and of these 4 kinases 36 showed at least a > 60-fold degree of selectivity for DNA-PKcs. In A549 cells **36** inhibits DNA-PKcs autophosphorylation at Ser2056 (IC_50_ = 90 nM); furthermore its selectively was also repeated in cells, with **36** showing a >90-fold selectivity for DNA-PKcs over ATM, ATR, mTOR, and three PI3K isoforms (α, β, and δ), with a 10-fold selectivity increase over PI3Kγ. The selectivity of **36** was compared to other notable DNA-PKcs inhibitors (including **37**) which all had at least one secondary target with <10-fold selectivity. **36** was able to enhance DNA damaging therapies such as radiotherapy, doxorubicin, and **1***in vitro* and *in vivo*. Due to its mechanism of acting through inhibition of NHEJ, as opposed to HR, **36** is differentiated from many of the current candidates for SL modulating drugs. This, combined with its potency and selectivity, makes it an interesting compound for further study.

M3814 (also known as nedisertib) (**37**, Figure 11B) is a potent selective DNA-PK inhibitor (DNA-PK IC_50_ ≤ 3 nM).^97^ Information on the development of this inhibitor has not been published, and **37** was first described in a patent filed by Merck.^98^**37** has been shown to enhance the antitumor effect of ionizing radiation in solid tumors and leukemia through inhibition of the NHEJ pathway, a common feature of selective potent DNA-PK inhibitors.^97,99,100^ This effect is much more pronounced in cancer cells that express WT-p53, thought to be due to overactivation of p53/ATM which causes a much higher concentration of p53 in comparison to radiation treatment administered alone, leading to premature cell cycle arrest and senescence. This enhancement of radiotherapy has also been observed in mice, where combination of **37** and radiotherapy caused complete remission.^97^ Due to these promising results *in vitro* and *in vivo*, four clinical trials are currently underway using **37**. These are listed in Table 3. To date, **37** is the only selective DNA-PK inhibitor involved in clinical development.

**Table 3:** List of Current Clinical Trials Involving Inhibition of DNA-PK

trial identifier	drug	phase	summary	status and accession date
NCT02316197	**37**	I	A first in human trial to assess the safety, efficacy, PK/PD properties, and tolerability of **37** in patients with advanced solid tumors or chronic lymphocytic leukemia. A secondary aim is to discover the correct dosing regime for **37** for future studies.	Completed (no results posted)
				December 12, 2014
NCT02516813	**37**	I	A trial to assess the safety, efficacy, and tolerability of **37** in combination with radiotherapy and chemotherapy in patients with solid tumors.	Recruiting
	Radiotherapy cisplatin			April 6, 2015
NCT03770689	**37**	I/II	The purpose of this study in phase Ib is to assess the maximum tolerated dose of **37** in combination with capecitabine and radiotherapy in participants with locally advanced rectal cancer. In Phase II the study seeks to evaluate the efficacy of **37** in terms of pathological complete response/clinical complete response in combination with capecitabine and radiotherapy, in comparison with a placebo and capecitabine/radiotherapy administered as monotherapies.	Recruiting
	Capecitabine radiotherapy			December 10, 2018
NCT03724890	**37**	I	A study to assess the maximum tolerated dose for phase II studies of **37** in combination with avelumab with or without radiotherapy in participants with advanced solid tumors.	Recruiting
	Avelumab radiotherapy			October 30, 2018
NCT01353625	**39**	I	A first in human trial to assess the safety and efficacy of **39** in participants with advanced solid tumors and hematologic malignancies who are unresponsive to standard of care treatments. The study also seeks to determine optimum dosages for later clinical trials and to assess the bioavailability of tablet and capsule formulations.	Active, not recruiting
				May 13, 2011
NCT01421524	**39**	I	A trial to assess the safety and efficacy of **39** in participants with advanced solid tumors, non-Hodgkin’s lymphoma, or multiple myeloma who are unresponsive to standard of care treatments, and to assess optimum dosing regiments for later clinical trials.	Active, not recruiting
				August 23, 2011

**Figure 12:**
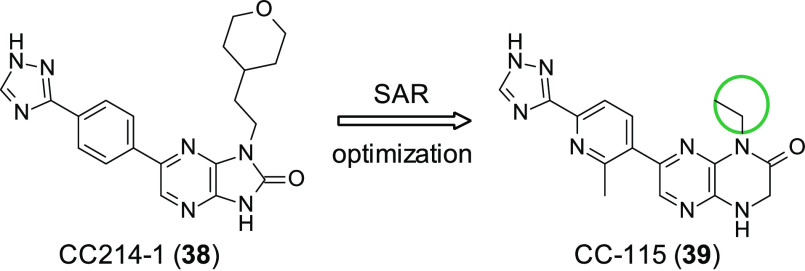
Optimization of mTOR inhibitor CC214-1 (**38**) leading to the discovery of mTOR/DNA-PK dual inhibitor CC-115 (**39**). Small substituents in the N1/N4 position resulted in the maintenance of high potency but improved PD/PK properties.

Mortensen et al. attempted to discover novel selective mTOR inhibitors through SAR studies of a series of 4,6- or 1,7-disubstituted-3,4-dihydropyrazino[2,3-*b*]pyrazine-2(1*H*)-ones, and in doing so, they discovered the potent dual mTOR/DNA-PK inhibitor CC-115 (**39**, Figure 12).^101^ Previous studies had identified CC214-1 (**38**, Figure 12) which displayed good on-target potency (mTOR IC_50_ = 2 nM); however it showed poor PK properties, showing negligible oral bioavailability. A focused SAR approach featuring 2-methyl-6-(1*H*-1,2,4-triazol-3-yl)pyridin-3-yl substituted analogues allowed the identification of small substituents in the N1/N4 position, which kept the excellent potency of the early compounds of the series while improving the PK profile. This culminated in the identification of **39** (mTORIC_50_ = 21 nM) as a clinical candidate. The DNA-PK activity of **39** was identified in separate work conducted by Tsuji et al., where inhibition of autophosphorylation of DNA-PK led to the blocking of DNA-PK facilitated NHEJ.^102^ In addition to **39**’s activity against mTOR and DNA-PK it was found to inhibit ATM, which as stated earlier is thought to have an SL relationship with DNA-PK; this interaction appears to have been confirmed as **39** is more active in ATM deficient cells, suggesting synthetic lethality between DNA-PK inhibition and ATM deficiency. This in combination with antitumor activity seen in solid tumor and hematopoietic cell lines and apoptosis/antiproliferation in various cell lines has lent support to **39** being explored as a clinical candidate. To date, **39** is featured in two clinical trials, which are reported in Table 3.

## 4 WEE1 Inhibitors

WEE1 is a kinase involved in cell cycle progression, where it prevents entry into mitosis in response to DNA damage. Furthermore, it has been implicated in the scheduling of cell division and HR repair.^103^ WEE1 prevents entry into mitosis through regulation of the G/M and S checkpoints through the phosphorylation of CDK1 and CDK2.^104^ As stated in this review, in the event of DNA damage, ATR or ATM pathways can be activated: ATR activation (Figure 3) results in the phosphorylation of CDK1, which leads to activation of checkpoint regulators of which WEE1 is one, which phosphorylates and inactivates the CDK1-cylin B complex on Tyr15, causing cell cycle arrest in G2.^105^ This halting of the cell cycle is preferential for cancer cells, as DNA damage is not repaired before replication in the S phase; therefore mutations can occur that favor proliferation, encouraging cancer growth. As the cancer cells are reliant on disruption of the apoptosis process, it is thought that the G2 checkpoint is vital for their survival, thereby presenting WEE1 as a promising target for cancer therapy. It is thought that healthy cells, containing fewer DNA breaks, will not be as affected by the disruption of the G2 checkpoint, and therefore this treatment would be considered somewhat selective for cancer cells.

WEE1 is highly expressed in numerous cancer types, including glioblastoma,^106^ breast cancer,^107^ leukemia,^108^ and others. Additionally, high levels of WEE1 expression have been observed in the event of severe DNA stress in various cancer types correlating with a poor prognosis.^109,110^ The cancer types that highly express WEE1 are thought to be extremely reliant on the G2 halting of the cell cycle, making them an attractive target for WEE1 inhibition.

**Figure 13:**
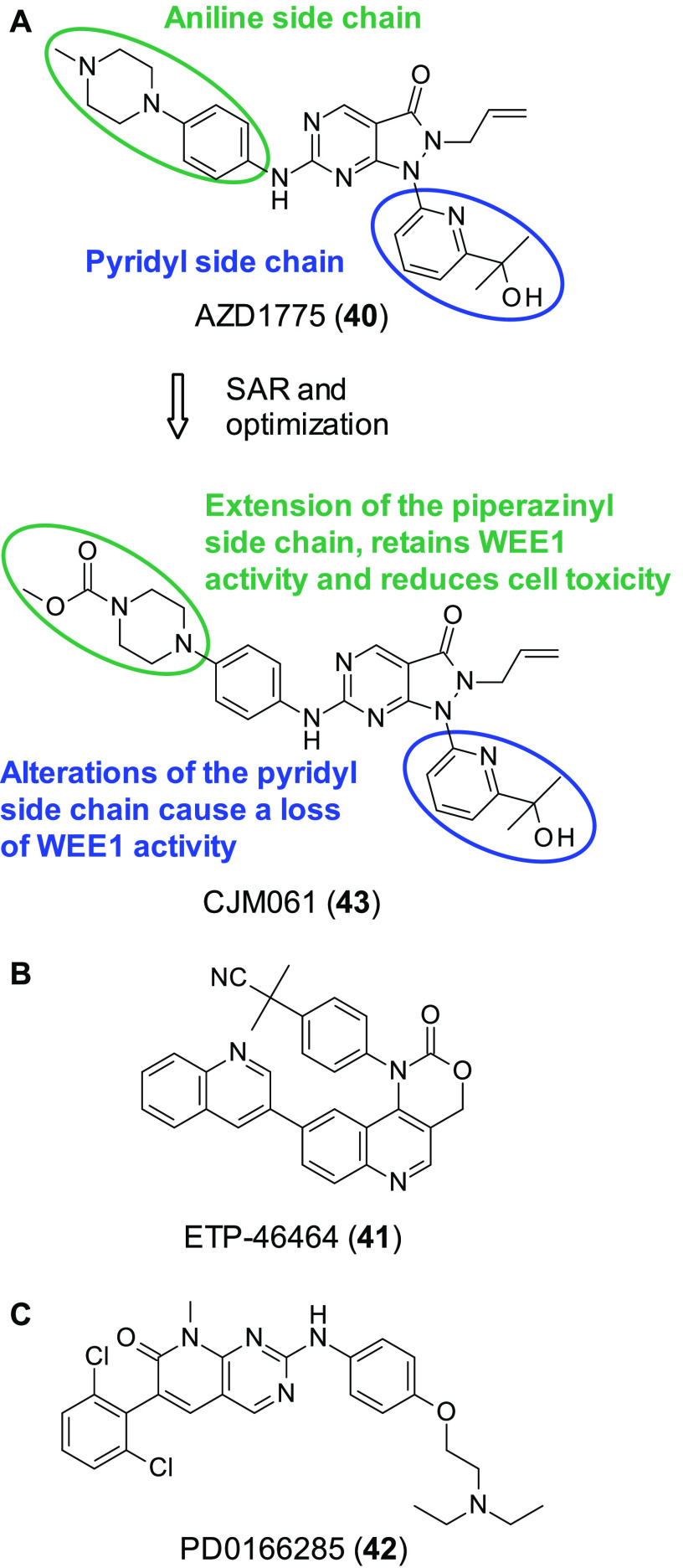
(A) SAR and optimization of WEE1 inhibitor **40** to **43**. (B) Structure of ATR inhibitor ETP-46464 (**41**). (C) Structure of first WEE1 inhibitor PD0166285 (**42**).

Synthetic lethality has reported between WEE1 and ATR inhibitors. This was achieved through the combination of the WEE1 inhibitor AZD1775 (formally MK-1775) (**40**, Figure 13A) and two ATR inhibitors **11** and ETP-46464 (**41**, Figure 13B).^111^ In this study synergistic killing of cancer cells from various tissues was observed, but untransformed cell remained unaffected. Through mechanistic studies using reversible inhibition of WEE1/ATR it was revealed that inhibition occurred in the G2/M phase. Additionally, through live cell imaging, it was seen that inhibition of WEE1 and ATR caused cells to enter mitosis, and cells with overly damaged DNA underwent mitotic catastrophe.

SL has been observed between WEE1 and TP53 mutants. TP53 encodes for the tumor suppressor p53 and is the most commonly mutated gene within human cancer and generally is thought to be associated with poor prognosis.^112^ As the mutations cause a loss of function, TP53 mutations cannot be directly targeted; therefore SL through WEE1 provides an opportunity to drug a previously undruggable target. As cells with TP53 mutations lack an effective G1 checkpoint, they become over-reliant on the G2 checkpoint; therefore inhibition of WEE1, which acts in the G2 checkpoint, is thought to cause SL. This has been demonstrated in TP53 mutant colorectal carcinoma where, after exposure to radiation, inhibition of WEE1 with the preclinical inhibitor PD0166285 (**42**, Figure 13C) caused the cells to evade G2 arrest and prematurely enter mitosis.^113^ Furthermore, **40** has shown substantial improvement in carboplatin treatment of advanced solid tumors with p53 mutants in phase I and phase II trials.^114,115^

Additionally, in a screen for SL partners for CHK1, WEE1 has been identified as a possible SL partner. Combination studies of **40** and the CHK1 inhibitor PF-00477736 in brain, ovarian, colon, and prostate cancer cells confirmed synergism between WEE1 and CHK1, independent of p53 status.^116^

A number of WEE1 inhibitors have been synthesized to date, with some in clinical studies. Here we will touch upon some of the most prominent inhibitors in the field.

The first WEE1 inhibitor developed was **42** and was identified through use of a HTS looking to find WEE1 inhibitors. **42** is a highly potent ATP competitive WEE1 inhibitor (IC_50_ = 24 nM) and was able to inhibit CDC2 at Tyr15 (a direct substrate of WEE1) in cells at 0.5 μM.^113^ Furthermore, it was able to sensitize cells to radiation therapy, with this effect being more pronounced in p53 deficient cells. However, **42** is not a selective inhibitor and shows IC_50_ < 100 nM for CDK1, MYT1, c-SRC, EGFR, FGFR-1, and PDGFR-β. **42** has been used as a tool compound for examining the effect of WEE1 inhibition in numerous cancers such as colon, lung, and melanoma, among others;^105^ however, its lack of selectivity has meant its usefulness beyond this is quite limited.

**40** was originally identified by Hirai et al. through use of a HTS; initial hits were optimized through SAR to yield the orally available selective potent small molecule **40** (Figure 13A) (WEE1 IC_50_ = 5.2 nM).^117^ A linear relationship between IC_50_ and ATP concentration indicated that **40** acted in an ATP competitive manner. To evaluate selectivity, **40** was tested in a kinase screen, and only 8 of the 223 kinases were inhibited >80% when treated with 1 μM of **41**, demonstrating remarkable selectivity for an ATP competitive kinase inhibitor. In cells WEE1 inhibition was observed through **40** inhibiting phosphorylation of CDC12 at Tyr15.

Furthermore, **40** abrogates the G2 DNA damage checkpoint and in combination with the DNA damaging agents gemcitabine, carboplatin, and cisplatin caused apoptosis in p53 deficient cells. **40** has also been observed to induce apoptosis and inhibit proliferation as a monotherapy in numerous cell types (acute lymphoblastic leukemia cells, lung cancer cells, and colorectal cancer cells among others).^118−120^ Furthermore, *in vivo* it was seen to inhibit tumor growth without perceived toxicity. As previous stated in the review, **40** has entered numerous phase I and II clinical trials and is the most established WEE1 inhibitor in the field.

As little SAR data exist for **40**, Matheson et al. synthesized a series of analogues to evaluate which structural features are necessary for successful WEE1 inhibition.^121^ The analogues that inhibited WEE1 in the same nM range had lessened cytotoxicity when administered as a single therapy in comparison to **40**, and synergism was observed in combination with cisplatin in medulloblastoma cells. CJM061 (**43**, Figure 13A) was the most active from this analogue series (WEE1 IC_50_ = 2.8 nM) and its development can be seen in (Figure 13A).

These promising compounds, especially **40**, show the potential for WEE1 inhibitors, with at least three confirmed SL relationships. Other than **40** no other WEE1 inhibitor is in clinical development. Should it prove to be unsuccessful in these clinical trials, more work is needed to fully make use of this attractive target for cancer therapy.

## 5 CDK12 Inhibitors

CDK12 was first identified when investigating the cell cycle regulator CDC2.^122^ While it has structural similarities to other members of the CDK subfamily, which play a role in cell cycle regulations, CDK12 is a transcription kinase involved in the transcription of genes involved in DNA repair,^123^ regulating specific genes that respond to stress, heat shock, and DNA damage.^124^ CDK12 presents a great opportunity to test SL due to the high amount of genomic alterations seen in numerous cancers including high-grade ovarian carcinoma,^125,126^ HER-2 positive breast cancer,^127^ and lung adenocarcinoma,^128^ resulting in loss of function. To date, SL pairs for CDK12 have been identified as PARP,^129^ MYC,^130^ and EWS/FLI.^131^

**Figure 14:**
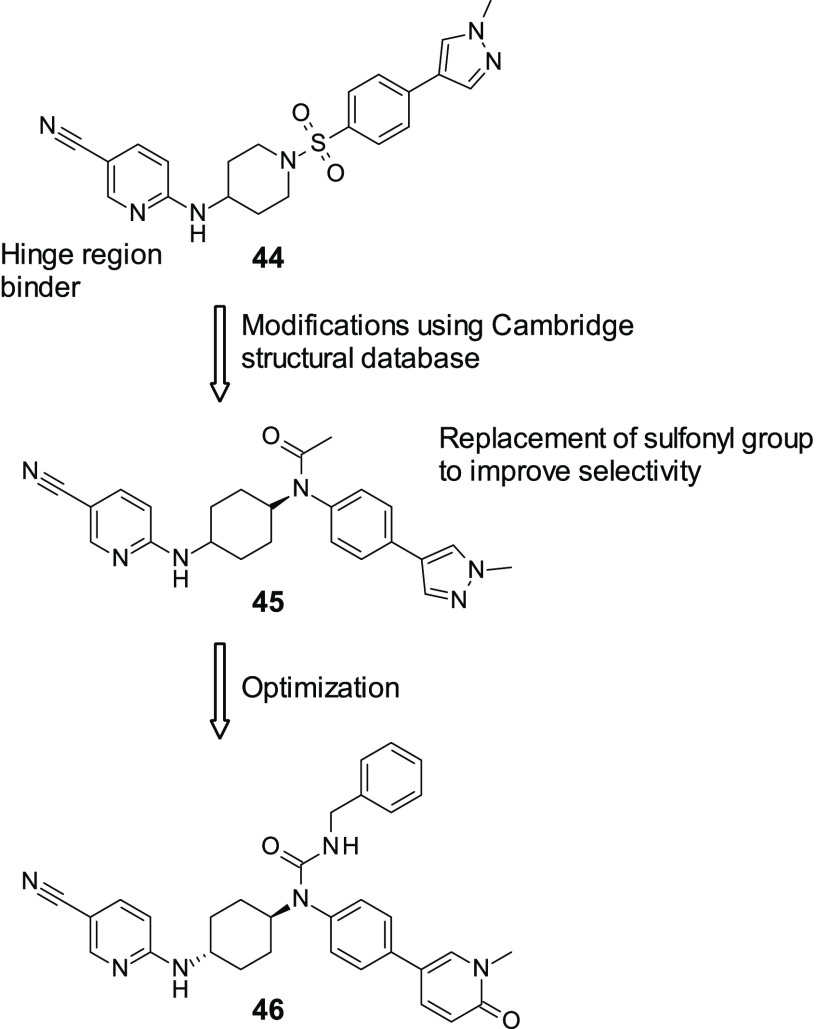
Optimization of CDK12 inhibitor **44** to **46**.

CDK12 mutations have been shown to sensitize cancers to traditional DNA damaging agents;^132^ therefore CDK12 inhibitors have gained traction in recent years as a way of implementing small molecule-induced synthetic lethality. A group within Takeda has developed a series of potent and selective CDK12 inhibtors,^133^ which also hit CDK13, another potential modulator of DNA repair. A modest initial CDK12 inhibitor (**44**, Figure 14) was identified through a HTS (CDK12 IC_50_ = 0.36 μM). While it demonstrated good selectivity over other related kinases within the CDK subfamily, it also showed activity against CDK2, which is involved in healthy regulation of the cell cycle; therefore, for **44** to be useful, it would need to be optimized further. Through SAR studies, they discovered that, first, the aminopyridyl moiety was binding in the hinge region of CDK12 and could not be modified. This is a fairly typical moiety for kinase inhibitors. Second, it was discovered through the same SAR studies that at the 5-position of the pyridine ring it is likely that an electron-withdrawing group is required. Through later docking simulations of **44** in both CDK2 and CDK12, it was proposed that the oxygen atom in the sulfonyl moiety interacted with Lys89 in CDK2, which is not conserved in CDK12. This was therefore changed, utilizing experimentally derived conformations from the Cambridge Structural Database, to either a *tert*-amide or a *tert*-sulfonamide. Of these, the *tert*-amide showed better selectivity for CDK12 over CDK2 (compound **45**, Figure 14). Some final modifications, such as the introduction of a benzyl group and small modifications to improve physiochemical properties, resulted in the highly selective inhibitor, with it only showing inhibition of over 80% at 1 μM in 3 of the 441 kinases present in the kinome screen. **46** (Figure 14) (CDK12 IC_50_ = 0.052 μM) was shown to inhibit CDK12 phosphorylation of SER2 in SKBR2 cells (pSer2 IC_50_ = 0.195 μM). This compound therefore presents a valuable tool in probing SL or could serve as a starting point for further drug discovery programs investigating SL.

Other CDK12 and CDK13 inhibitors have emerged within the same period, in work completed by Zhang et al.^134^ Previous work conducted by the group yielded the CDK7 inhibitor THZ1 (**47**, Figure 16A).^135^**47**, while being active against CDK7 (IC_50_ = 3.2 nM), also showed activity against CDK12 (IC_50_ = 864 nM) and CDK13 (IC_50_ = 225 nM) and was therefore a starting point for further drug discovery. It was reasoned initially that alteration of the acrylamide moiety would divert the binding toward Cys1039 of CDK12. Through SAR studies that are yet to be reported, the compound THZ531 (**48**, Figure 16A) was synthesized. **48** has shown IC_50_’s in biochemical assays of 158 nM and 69 nM for CDK12 and CDK13, respectively, and has a more than 50-fold level of selectivity over the other members of CDK family. Interestingly, when the electrophilic acrylamide was replaced by a moiety incapable of covalently binding, propylamide, the activity of the compound dramatically reduced, suggesting that the ability to covalently bind is necessary for preserving CDK12 and CDK13 activity. Its selectivity over the wider kinome is good, with none of the other 211 kinases tested showing an activity of over 55%.

**Figure 15:**
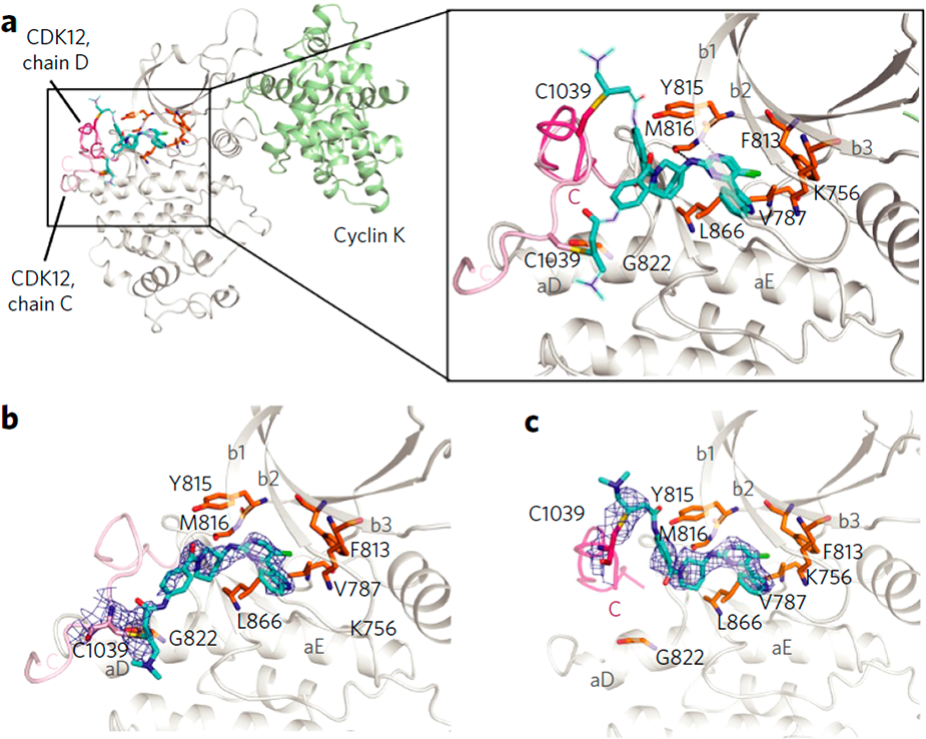
Cocrystallization of **48** with CDK12 adapted from Zhang et al.^134^ (a) **48** binds to M816 in the kinase hinge region and connects to Cys1039 in two conformations via the compound’s flexible linker. Solvent-exposed regions of **48** with poor electron density are represented by thin sticks. (b) Omit map contoured at 2.5σ for **48** bound to CDK12 chain C. (c) Omit map contoured at 2.5σ for **48** bound to CDK12 chain D. Reproduced with permission from Springer Nature, Nature Chemical Biology, Covalent targeting of remote cysteine residues to develop CDK12 and CDK13 inhibitors, Tinghu Zhang, Nicholas Kwiatkowski, Calla M. Olson, Sarah E. Dixon-Clarke, Brian J. Abraham, Ann K. Greifenberg, Scott B. Ficarro, Jonathan M. Elkins, Yanke Liang, Nancy M. Hannett, Theresa Manz, Mingfeng Hao, Bartlomiej Bartkowiak, Arno L. Greenleaf, Jarrod A. Marto, Matthias Geyer, Alex N. Bullock, Richard A. Young, Nathanael S. Gray10.1038/nchembio.2166PMC503307427571479,^134^ Copyright 2019 Springer Nature.

**48** has been cocrystallized with CDK12. This has presented some useful binding information, which could serve others in the field, in the pursuit of novel CDK12 inhibitors (Figure 15). Two CDK12-cycklin K complexes were found, each bound to **48** in a different rotamer. These crystallization studies revealed that a labile αK helix can be displaced from CDK12 allowing binding through the linker of **48** with Cys1039. The aminopyrimidine of **48** was found to form two hydrogen bonds to the backbone of M816, with the pendant 3-indolyl and the piperidine groups involved in hydrophobic interactions. The binding of the secondary amide was shown to have two different conformations that caused the solvent exposed groups to pack either against the N-lobe β strand or C-lobe αD helix. The resolution for the exposed groups was too poor to be defined apart from the position of Cys1039. Interestingly Cys312, the equivalent sulfhydryl to Cys1039 in CDK7, was further away from the binding region, suggesting poorer binding for **49** against CDK7 in comparison to CDK12. **49** was able to induce apoptosis in Jurkat cancer cells, in addition to being able to inhibit transcriptional regulation, reducing DNA damage repair and superenhancer gene expression. **49** has been shown to demonstrate a synergistic effect with PARPi’s in Ewing sarcoma in both cells and mouse models, as it was seen that the Ewing sarcoma cell models were particularly sensitive to CHK12 inhibition. Furthermore, the expression of the tumor specific oncogene EWS/FLI shows SL with CHK12 inhibition. The inhibition of CHK12 was also seen to impair DNA damage repair in Ewing sarcoma cells.^131^

**Figure 16:**
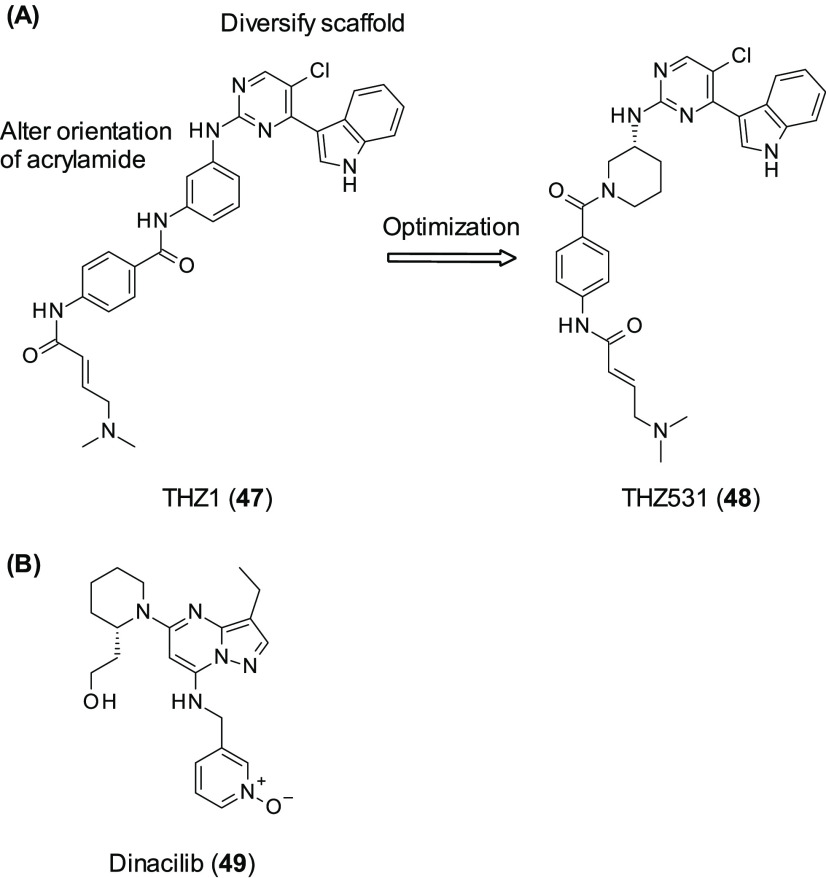
(A) Optimization of CDK inhibitor THZ1 (**47**). (B) Structure of CDK inhibitor dinaciclib (**49**).

A known CDK inhibitor, dinaciclib (**49**, Figure 16B),^136^ whose primary targets are CDK1, CDK2, CDK5, and CDK9, has shown a documented response in breast cancer^137^ and in recent years has also been shown to inhibit CDK12. While showing a higher level of promiscuity and also hitting cell cycle kinases within the family, **49** is the most potent CDK12 inhibitor to date, with an IC_50_ of 40–60 nM in biochemical assays.^138^ In cell-based assays, **49** showed phenotypic responses typical of CDK12 inhibition, including gene repression of multiple genes involved in HR, whose expression is thought to correlate with CDK12 expression.^139^ This was achieved with minimal disruption to the cell cycle, indicating CDK12 inhibition as the cause of this observation. Due to the dysregulation of HR influencing genes, it was reasoned that **49** would be able to sensitize triple-negative breast cancer cells to PARPi. This was observed through the combination of **49** with the known PARP inhibitor veliparib, which showed a 2.5- to 12.5-fold increase in its activity as a single-agent treatment, demonstrating a synergistic SL effect. This impairment of HR by **49** was mirrored through knockout of CDK12, showing the same phenotype. Not only was **49** capable of showing a synergistic effect with PARPi’s, it was also able to resensitize triple-negative breast cancer cells that had become resistant to PARP inhibition to **1**. These results seem to indicate that the use of **49** can cause an increase in the effectivity of PARP inhibition even if a partial response is seen with the PARPi being administered as a monotherapy.

CDK12’s progress as a target has meant that the idea of utilization of CDK12’s synthetic lethality has moved on to clinical trials. While there is not a great abundance of trials directly using CDK12 inhibitors, other potential SL partners are being modulated in CDK12 deficient cancers, thus giving an SL effect. If these worked, it opens the door for chemically induced small-molecule lethality. The trials are summarized in Table 4. One CDK12 inhibitor has entered clinical trials, and a number of selective CDK12 inhibitors are currently in preclinical studies. CDK12 inhibitors have the potential to be useful in the application of SL to cancer therapy.

**Table 4:** Clinical Trials Involving Loss of CDK12 Function

trial identifier	drug	phase	summary	status and accession date
NCT01434316	Veliparib (PARPi) and **49**	I	Studying the safety profile of veliparib and **49** in participants with advanced solid tumors.	Recruiting
				September 14, 2011
NCT04272645	Abemaciclib and atezolizumab (both PD-L1 immunotheraputics)	II	Studying the effectivity and safety of abemaciclib and atezolizumab in participants with metastatic castration-resistant prostate cancer with and without “CDK12 loss” mutation.	Withdrawn (coordinating site change)
				February 17, 2020
NCT03570619	Nivolumab and ipilimumab (both PD-L1 immunotheraptics)	II	Studying the efficacy of nivolumab/ipilimumab in combination and nivolumab as a monotherapy in participants with castration-resistant metastatic prostate carcinoma or other solid tumor histologies, with CDK12 loss of function.	Recruiting
				June 27, 2018
NCT04104893	Pembrolizumab (PD-L1 immunotheraptic)	II	A study to assess the activity and efficacy of pembrolizumab in participants with progressive metastatic castration-resistant prostate cancer, characterized by a mismatch repair deficiency or biallelic CDK12 inactivation.	Recruiting
				September 26, 2019

## 6 PARP and RAD51

RAD51 is a member of the RAD52 epistasis group, which is made up of RAD50, RAD51, RAD52, RAD54, RAD55, RAD57, RAD59, MRE11, and XRS2.^140^*In vitro* studies have shown that RAD51 promotes homologous pairing and strand transfer reactions. Following treatment by DNA-damaging agents, RAD51 was seen to be upregulated, indicating its role in HR. Early on during HR, DSB or SSB results in the formation of a length of ssDNA. This strand is then paired with recombinases such as RAD51 and afterward is paired with a homologous duplex to form a DNA joint which is termed the D loop.^141^ RAD51 then goes on to promote an ATP-mediated strand exchange reaction by polymerizing on DNA, resulting in a helical filament. The formation of this is completed in two steps, nucleation and extension, promoting HR. RAD51 has also been shown to cause polymerization of dsDNA; however the reason for this has yet to be elucidated.^142^

**Figure 17:**
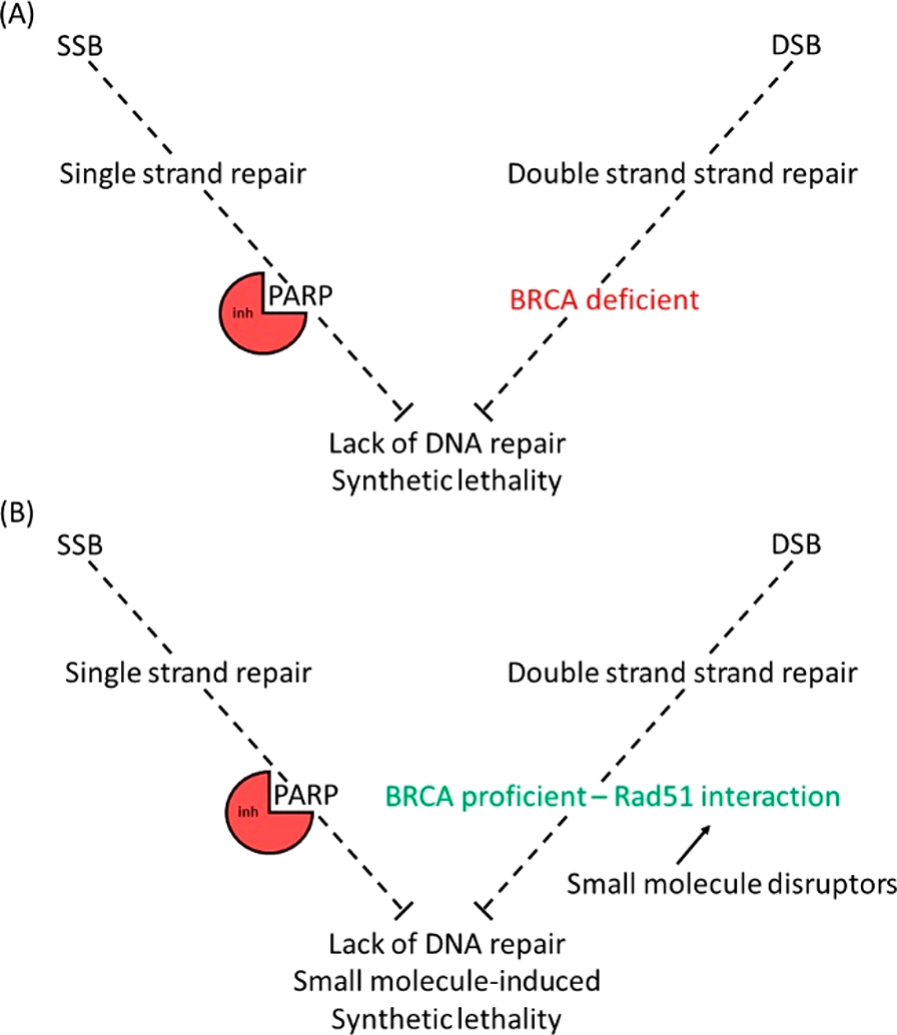
(A) PARP inhibitors triggering synthetic lethality in BRCA-deficient cells. (B) Proposed triggering of small molecule-induced synthetic lethality using PARP inhibitors in combination with RAD51-BRCA2 disruptors. Adapted from European Journal of Medicinal Chemistry, Vol. 165, Marinella Roberti, Fabrizio Schipani, Greta Bagnolini, Domenico Milano, Elisa Giacomini, Federico Falchi, Andrea Balboni, Marcella Manerba, Fulvia Farabegoli, Francesca De Franco, Janet Robertson, Saverio Minucci, Isabella Pallavicini, Giuseppina Di Stefano, S. Girotto, R. Pellicciari, A. Cavalli, Rad51/BRCA2 disruptors inhibit homologous recombination and synergize with olaparib in pancreatic cancer cells10.1016/j.ejmech.2019.01.00830660828,^143^ Page 81, Copyright (2019), with permission from Elsevier.

**Figure 18:**
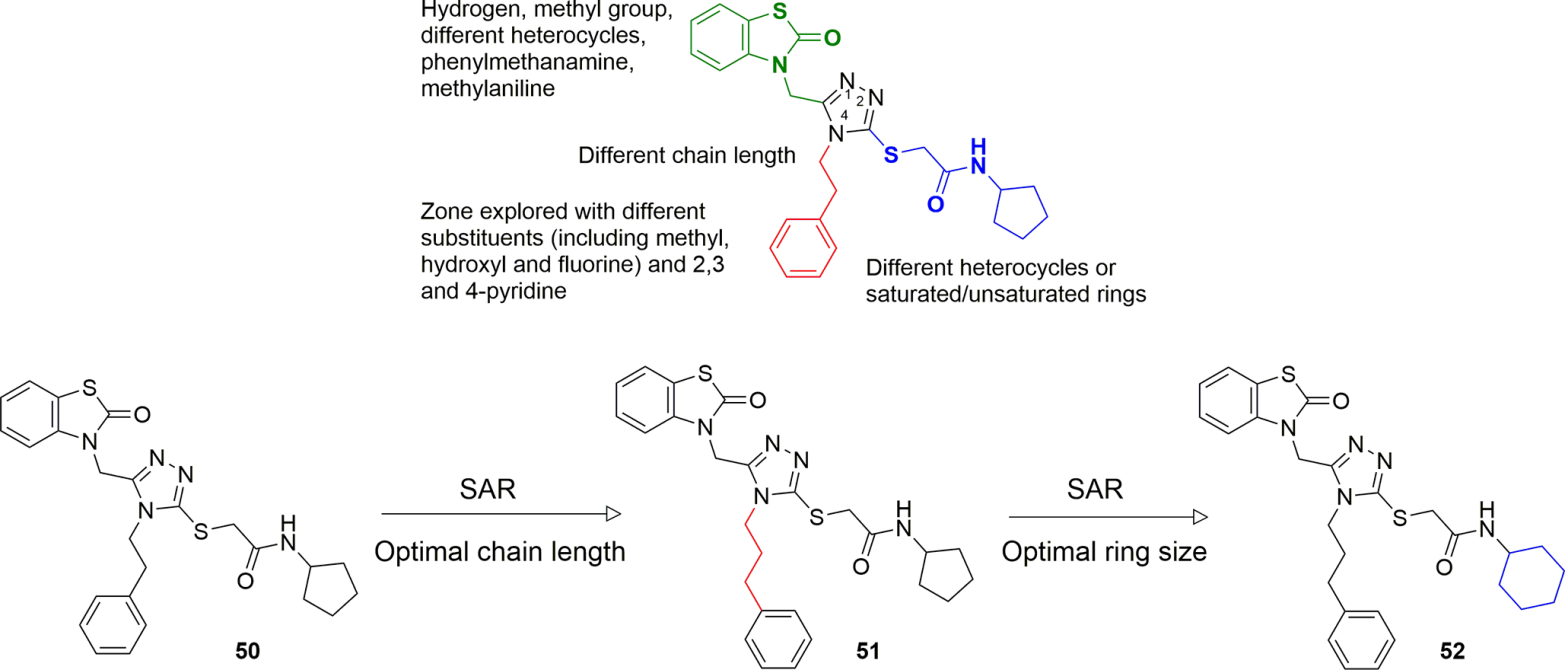
Depiction of the SAR strategy for the further development of **52**. This primarily focused on three areas within the molecule highlighted in green, red, and blue. The proposed moieties in these regions are shown in their accompanying text.

It has been hypothesized that interrupting BRCA2 and RAD51’s interaction would mirror the synthetically lethal effect seen in utilizing PARPi in BRCA2 deficient tumors^143^ (Figure 17). This would provide a shortcut to modulating the activity of BRCA2 which, to date, has been thought to be undruggable. While RAD51 inhibitors do exist,^144−146^ they have not been seen to interfere with the BRCA2–RAD51 interaction. For instance, structural data have shown that BRC4 (the fourth BRC repeat of BRCA2) binds to RAD51 in two hydrophobic binding pockets: one of these is critical for RAD51 multimerization (FxxA). Utilizing high-throughput docking, our group began to work developing modulators for this interaction. From the initial hit compounds identified from the HTS, **50** (Figure 18) was the best performing (EC_50_ = 53 ± 3 μM), and as such, an SAR program was initiated, at first investigating the optimal length of the alkyl chain between the triazole and the phenyl ring. From these studies, **51** (Figure 18) (EC_50_ = 25 ± 2 μM) which features a propyl phenyl ring was selected for further biological evaluation. **51** was tested in combination with **1** in two pancreatic cancer cell lines: Capan-1 which lacks functional BRCA2; BxPC-3 which is BRCA2-positive. Unsurprisingly as a monotherapy, **1** was more effective in the Capan-1 cells, due to the lack of BRCA2. Upon combination with **51**, no effect was seen in the Capan-1 cells; however, in the BxPC-3 cells, a synergistic effect was seen between **51** and **1**, suggesting small molecule-induced synthetic lethality. **51** was tested on the same cell lines that had undergone prior treatment by cisplatin: it demonstrated no effect in the BRCA2-negative cells, whereas in the BRCA2-positive cell line, a small increase in a phenotypic indicator for DNA damage was observed, confirmed by silencing of RAD51 which demonstrated a similar effect to **51**. This work could potentially provide a way of inducing SL in patients who lack the BRCA1/2 mutation, increasing the scope of PARPi’s.

Further SAR optimization of **51** was undertaken.^143^ These focused around adjusting the chain length of the N4 nitrogen and introducing substitutes on the phenyl ring. Other modifications included changes at the C3 position of the triazole, introducing a series of heterocycles, saturated and unsaturated rings with differing links. Lastly, changes occurred at the C5 position of the triazole, changing the heterocycle present at the position or removing this substituent entirely, replacing it with either a methyl group or a proton; these alterations are summarized in Figure 18. Modifications at the C5 and N4 positions were not well-tolerated, with a drop in activity usually observed. A minor tweak, changing the cyclopentyl group for a cyclohexyl group, resulted in the best compound from the series, compound **52** (Figure 18) (EC_50_ = 8 ± 2 μM).

**Figure 19:**
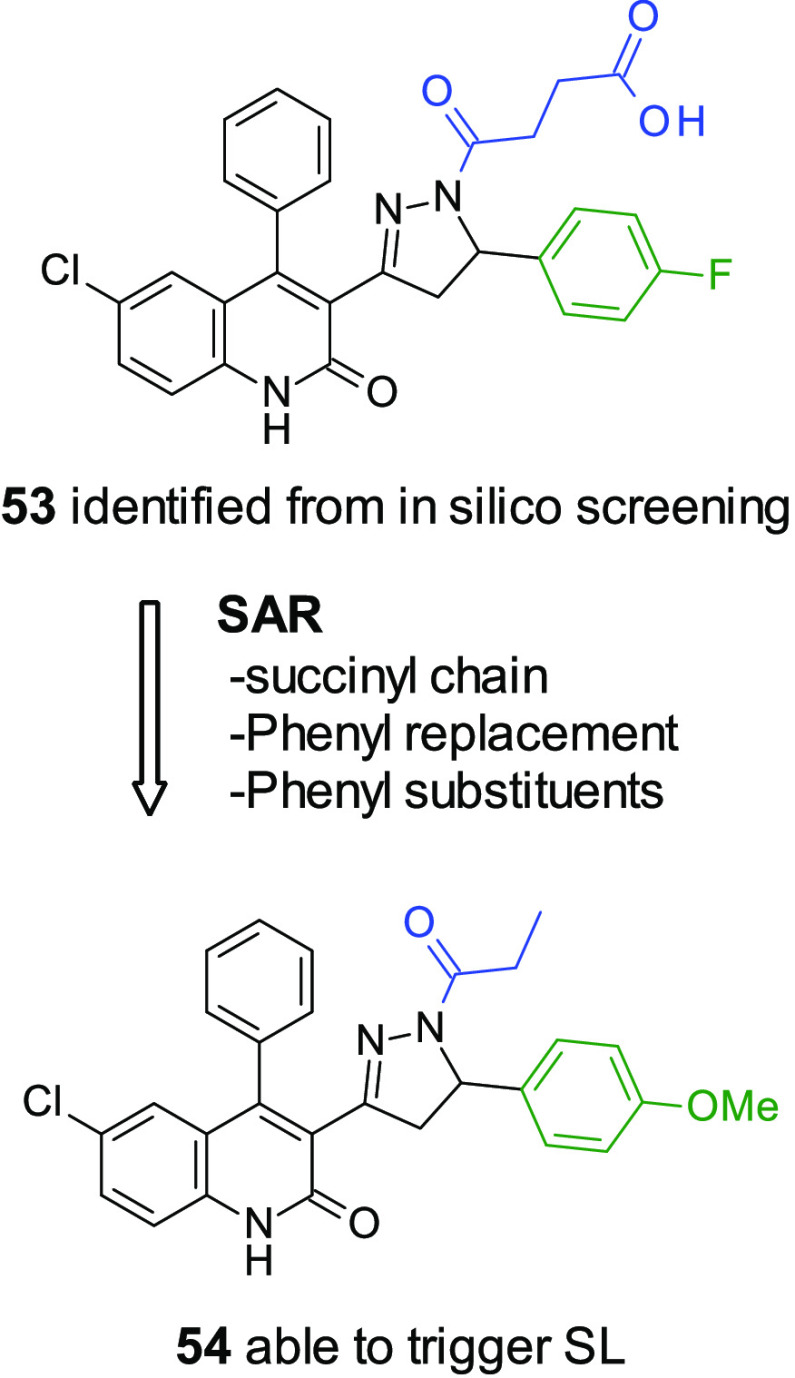
Optimization of **53** to **54**. All compounds from this series were tested as racemic mixtures after both enantiomers of **53** showed the same biochemical activity and binding mode.^147^

**52** inhibited the RAD51–BRCA2 interaction, preventing RAD51 function in cells. Furthermore, it significantly improved the function of **1** in BRCA2-positive cells and increased the amount of DSBs when administered in combination with **1**. However, due to its low potency or the presence of a mutant from p53 that prevents apoptosis, true synthetic lethality could not be observed, and showing further optimization was needed.^143^ In order to increase diversity within the field of Rad51–BRCA2 inhibitors, our group ran a second binding screening at the other binding site (LFDE).^147^ Mutations in this binding pocket are associated with cellular lethality, in addition to the failure of RAD51 assembly in nuclear foci at the point of DNA breaks *in vivo*, suggesting a key role in RAD51’s function.^148^ Of the compounds tested, the dihydroquinolone pyrazoline derivative **53** (Figure 19) (EC_50_ = 16 ± 4 μM) was the most active, and an SAR program was initiated around this compound (this work will be published in a subsequent paper), eventually yielding compound **54** (Figure 19) (EC_50_ = 19 ± 1 μM). Like **52** from the previous work, **54** was able to bind to RAD51 inhibiting its function, but unlike **52**, **54** was able to trigger SL in a dose response manner by impeding HR in pancreatic cancer cells that expressed BRCA2. However, due to **54**’s poor solubility, it is unlikely to be able to be tested it *in vivo*, and therefore further optimization is required before these class of compounds can realize their potential. This work is ongoing and demonstrates one of the first programs targeting small molecule-induced synthetic lethality and a way to expand the use of PARPi’s beyond BRCA1/2 deficient cancers, increasing their usefulness.

**Figure 20:**
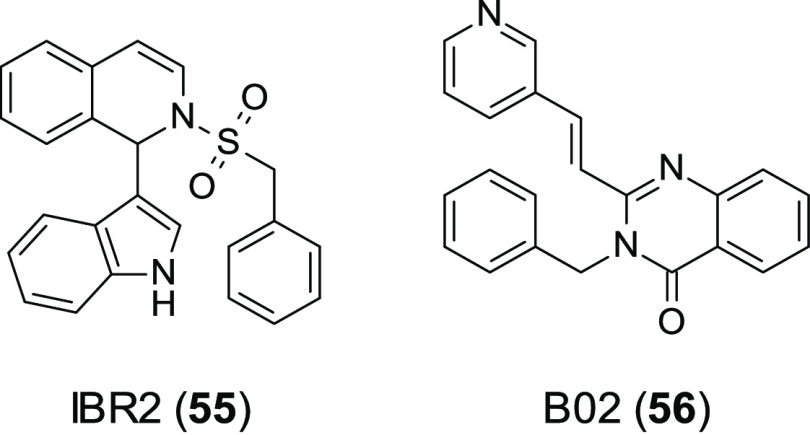
Examples of Rad51 inhibitors reported in the literature.

A number of other RAD51 inhibitors have been reported in the literature,^144−146,149−156^ the most noteworthy of which are shown in Figure 20. Only IBR2 (**55**, Figure 20) (RAD51 IC_50_ not reported) and BO2 (**56**, Figure 20) (RAD51 IC_50_= 27.4 μM) have been directly linked to small molecule-induced synthetic lethality.^155,157^**55** was observed to synergize with multiple drugs (notably imatinib, regorafenib, EGFR inhibitors (including erlotinib, gefitinib, afatinib, and osimertinib), and vincristine) with differing molecular targets, which were not involved in the RAD51 mediated HR pathway. However, the combination of **55** with DNA-damaging agents such as cis-platin was not synergistic.^155^ In the same study **55** was compared to **56**, which was able to synergize with imatinib and vincristine, thereby opening the door for these two compounds to be explored for SL. At a recent conference, Maclay et al. reported a novel RAD51 inhibitor: CYT01B.^158^ Neither the structure of this compound nor the data associated with it have been published to date; however a patent has been released by the parent company Cytier Therapeutics, which appears to be indicative of the structure.^159^ CYT01B has been shown to cause DNA stress through DNA replication fork damage. Therefore, it was tested to see if CYT01B could sensitize cells to current therapeutics for the treatment of solid tumors. This was done through combination assays with CYT01B (concentration range of 20 nm to 5 μM) in three cell lines with six targeted agents, the results of which are summarized in Table 5. These studies, in particular the success seen with the platinum-based chemotherapy carboplatin, in addition to PARPi’s, seem to indicate a degree of synthetic lethality. Therefore, this compound should be monitored as it progresses through *in vivo* models and beyond. As little is known about the compound’s selectivity panel, it is impossible to say whether the observed SL phenotype is due to RAD51 inhibition; however, should this information come to light, it would no doubt be of interest to the field of not only RAD51 inhibitors but also small molecule-induced synthetically lethality.

**Table 5:** Summary of Cell-Based Combination Studies Featuring CYT01B

cell line	drug combination	response observed
ARPE19/HPV16 (HPV immortalized normal epithelial cell line)	V822 (ATRi) (concentration range of 39 nM to 2.5 μM) and CYT01B (20 nM to 5 μM)	Synergism observed at all concentrations.
	TDRL-505 (RPAi) (concentration range of 39 nM to 5 μM) and CYT01B (20 nM to 5 μM)	Antagonism observed at all concentrations.
	Bortezomib (proteasome inhibitor) (concentration range of 39 nM to 2.5 μM) and CYT01B (20 nM to 5 μM)	Synergism observed at all concentrations.
	Carboplatin (nonspecific chemotherapeutic) (concentration range of 156 nM to 10 μM) and CYT01B (20 nM to 5 μM)	Synergism observed at all concentrations.
	**1** (concentration range of 78 nM to 5 μM) and CYT01B (20 nM to 5 μM)	Synergism observed at all concentrations but stronger synergy than that of niraparib.
	Niraparib (**3**) (PARPi) (concentration range of 78 nM to 5 μM) and CYT01B (20 nM to 5 μM)	Synergism observed at all concentrations.
KYSE-70 (head and neck cancer cell line)	V822 (ATRi) (concentration range of 39 nM to 2.5 μM) and CYT01B (20 nM to 5 μM)	Synergy was observed with CYT01B at 39 nM, but an antagonistic relationship between V822 and CYT01B was seen at low and high concentrations.
	TDRL-505 (RPAi) (concentration range of 39 nM to 5 μM) and CYT01B (20 nM to 5 μM)	Weak synergy observed at 156 and 312 nM of CYT01B.
	Bortezomib (proteasome inhibitor) (concentration range of 39 nM to 2.5 μM) and CYT01B (20 nM to 5 μM)	Antagonism observed at all concentrations.
	Carboplatin (nonspecific chemotherapeutic) (concentration range of 156 nM to 10 μM) and CYT01B (20 nM to 5 μM)	Synergism observed at all concentrations.
	**1** (concentration range of 78 nM to 5 μM) and CYT01B (20 nM to 5 μM)	Synergism observed at all concentrations but stronger synergy than that of niraparib.
	Niraparib (**3**) (PARPi) (concentration range of 78 nM to 5 μM) and CYT01B (20 nM to 5 μM)	Synergism observed at all concentrations.
Daudi (Burkitt’s lymphoma cell line)	V822 (ATRi) (concentration range of 39 nM to 2.5 μM) and CYT01B (20 nM to 5 μM)	Antagonistic effect at high concentrations but additive at low.
	TDRL-505 (RPAi) (concentration range of 39 nM to 5 μM) and CYT01B (20 nM to 5 μM)	Antagonism observed at all concentrations.
	Bortezomib (proteasome inhibitor) (concentration range of 39 nM to 2.5 μM) and CYT01B (20 nM to 5 μM)	Antagonism observed at all concentrations.
	Carboplatin (nonspecific chemotherapeutic) (concentration range of 156 nM to 10 μM) and CYT01B (20 nM to 5 μM)	Synergism observed at all concentrations.
	**1** (concentration range of 78 nM to 5 μM) and CYT01B (20 nM to 5 μM)	Synergism observed at all concentrations but stronger synergy than that of niraparib.
	Niraparib (**3**) (PARPi) (concentration range of 78 nM to 5 μM) and CYT01B (20 nM to 5 μM)	Synergism observed at all concentrations.

## 7 RAD52 Inhibitors

**Figure 21:**
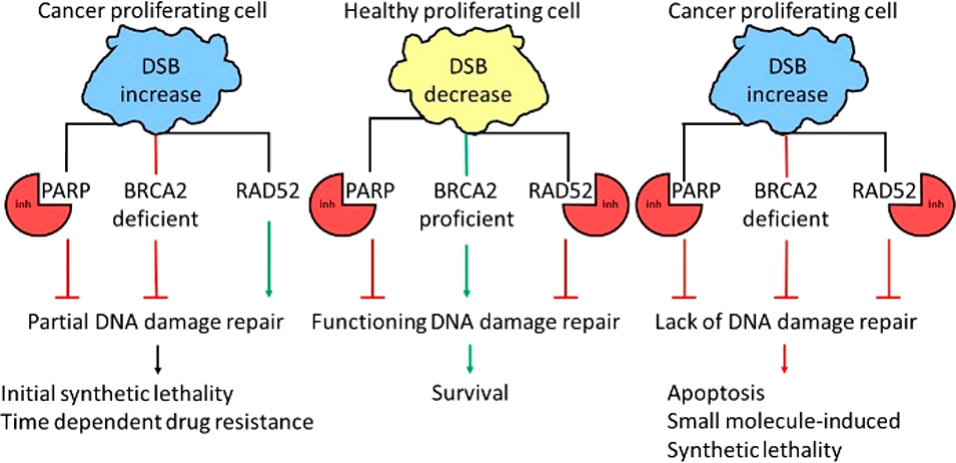
RAD52, SL interactions with PARP inhibitors. This figure shows classic SL with PARP inhibition in BRCA2 deficient-cancer cells. These are prone to acquired mutation. The implementation of RAD52 in combination with PARPi has no effect in BRCA2 proficient healthy cells; however, in BRCA2-deficient cancer cells, small-molecule-induced synthetic lethality occurs. Adapted from Cancers, 2019, Vol. 11, Issue (10), , Monika Toma, Katherine Sullivan-Reed, Tomasz Śliwiński, Tomasz Skorski, RAD52 as a Potential Target for Synthetic Lethality-Based Anticancer Therapies10.3390/cancers11101561PMC682713031615159,^162^ Page 1569, with use of the Attribution 4.0 International (CC BY 4.0) open access license.

The RAD52 protein is able to bind to ssDNA and plays a key role in repairing of single-strand and double-strand breaks.^160^ Within the HR response, it is essential in more simple organisms such as bacteria or yeast, where it acts in aiding RAD51 attachment to ssDNA. However, in more complex organisms such as animals this is largely completed by other proteins such as BRCA1/2. Indeed, knockout of RAD52 in mice does not cause a drop in viability or fertility of the mouse. However, upregulation of RAD52 can have a protecting effect on DNA from ionizing radiation, suggesting it is important to the HR process. Common synthetic lethality partners are BRCA1/2, which play a vital role in annealing RAD51 to ssDNA; however mutations in these proteins are quite common,^161^ and when this occurs, the cancer cell can compensate by utilizing RAD52 to perform the task instead. This is represented in Figure 21. This makes RAD52 an appealing target for cancer therapy, as it will only have key function in HR when BRCA1/2 are inactive: this will therefore have a selective effect on cancer cells, without influencing health cells.

One of the mechanisms of synthetic lethality proposed for both RAD52 and BRCA1/2 inhibition is that endonuclease/exonuclease/phosphatase family domains containing protein 1 (EEPD1) mediate DNA cleavage in the absence of BRCA1/2 and RAD52 and cause the production of toxic intermediates that trigger cell death.^163^ This theory is supported by the fact that suppression of EEPD1 in BRCA1/2- and RAD52-compromised cells causes a drop in the level of synthetic lethality.^162^ With RAD52 appearing to be a promising target for SL-based drugs, a few examples of small molecule inhibitor discovery programs are discussed below.

The first attempt to modulate RAD52 activity was not done through use of small molecules but rather use of an aptamer.^164^ This was achieved through use of the F79 aptamer that demonstrated activity in BRCA1/2 deficient cells but had no effect on HR in normal cells. F79 demonstrated SL in leukemia but also breast, pancreatic, and ovarian cells. *In vivo* tests in mice showed a lengthening of lifespan in mice with BCR-ABL1-positive leukemia. Lastly, RAD52 showed a synergistic effect with nonspecific chemotherapeutics, suggesting that, utilized together, the dose of the chemotherapeutic could be lowered, thereby reducing the risk of side effects. While not being investigated further, aptamers could play a key role in SL targets, specifically those in HR, as elsewhere aptamers have been reported to inhibit the DNA strand exchange of RAD51.^165^ Again, this work has not been expanded upon and could provide an untapped resource for the synthetic lethality.

**Figure 22:**
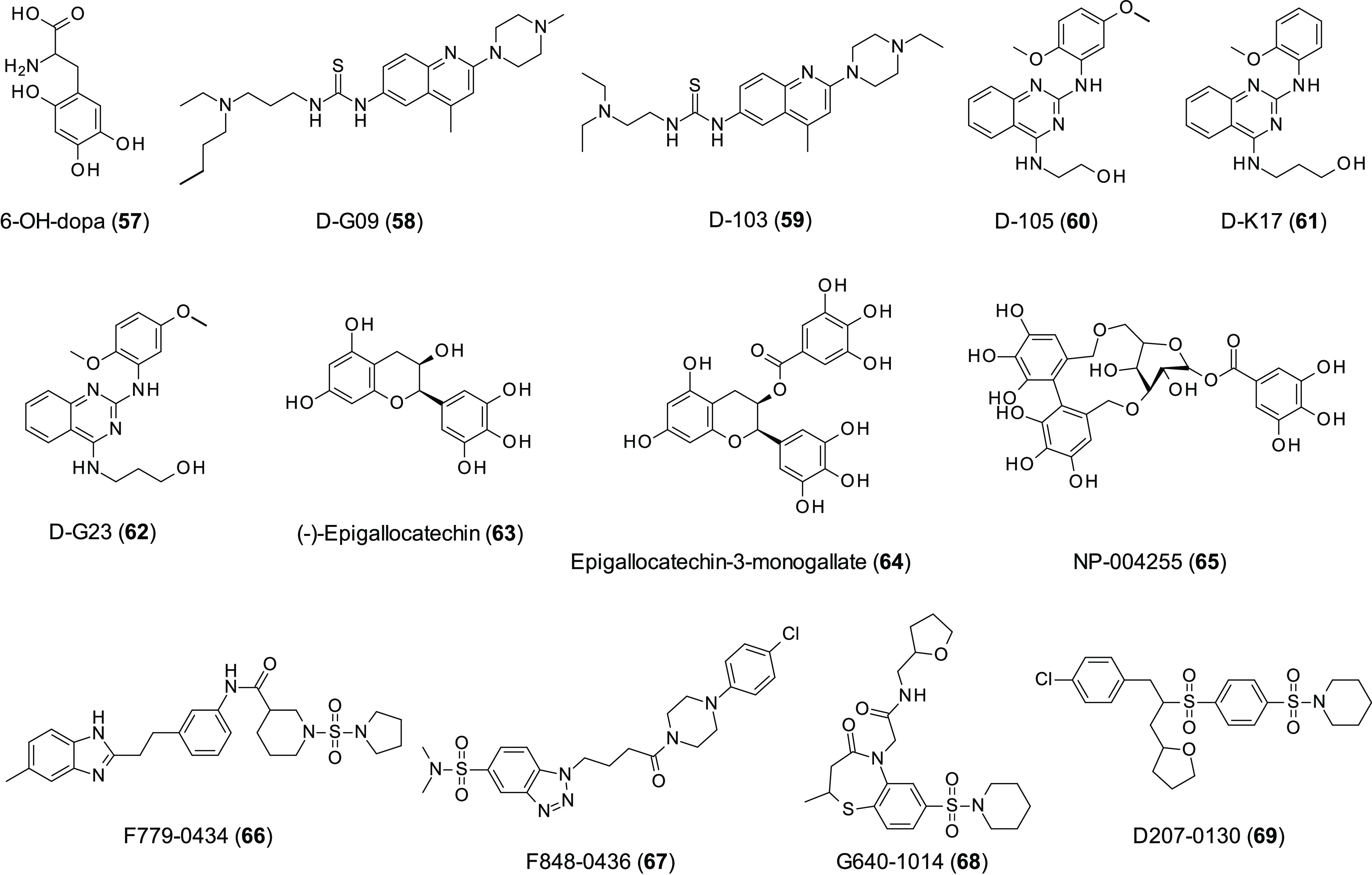
Structures of different RAD52 inhibitors.

6-OH-dopa (**57**, Figure 22) is a RAD52 inhibitor that was identified from a HTS of 18 304 drug-like compounds combined with 1280 from the Sigma Lopac collection. These compounds were screened through a high-throughput fluorescence polarization assay, and 10 compounds were identified that prevented RAD52 binding to ssDNA at a greater degree than 60%, at IC_50_ < 5 μM.^166^ Of these 10 compounds, only one was able to inhibit single-strand annealing in cells: **57**. It was later tested to see if it could inhibit HR. Predictably, it did not, due to RAD52’s weak role in healthy cells, therefore indicating some degree of selectivity for RAD52 over other key HR proteins. Furthermore, in biochemical assays, **57** was shown to have a significant degree of selectivity for RAD52 (1.1 μM) over RAD51 (not determined). **57** was also observed to halt proliferation in two cell lines deficient in BRCA1/2 (a Chinese hamster cell line and a pancreatic cancer cell line). In addition, separate studies also showed an increased level of apoptosis and DNA damage in BRCA1/2 cells. Despite being a promising compound, **57** is a dopaminergic derivative, which has been reported to heighten the chance of Parkinson’s disease by degenerating mitral neurons.^167^ Therefore, within cancer therapy, this is highly unlikely to be explored further. Huang et al. set out to develop small molecule inhibitors of RAD52 through use of a HTS.^168^ The hit compounds from this screen were tested for RAD52 selectivity, especially over RAD51, and in BRCA1/2 positive and deficient pancreas, ovarian, and triple negative breast cancer cells. Two hit compounds, D-G09 (**58**) and D-103 (**59**) (Figure 22), showed the expected phenotype for RAD52 inhibition in these cell lines, while another three compounds D-105 (**60**), D-K17 (**61**), and D-G23 (**62**) (Figure 22) showed activity against two of the cells lines but were of a structurally diverse scaffold, giving more opportunity for chemical space exploration. The best-performing compound of these, **59** (inhibits RAD52 ssDNA annealing at IC_50_ = 5 μM), was also tested in BRCA1/2-positive and -negative chronic myeloid leukemia cells, showing preferential inhibition of growth of the BRCA1/2-negative cells. Through SPR these compounds were confirmed to inhibit DNA, annealing through direct binding with RAD52 as opposed to DNA substrates. It was also demonstrated that **59** shows an absence of a nonspecific effect on RAD-51 foci production in response to cisplatin, reinforcing the selectivity of the compound.

**Figure 23:**
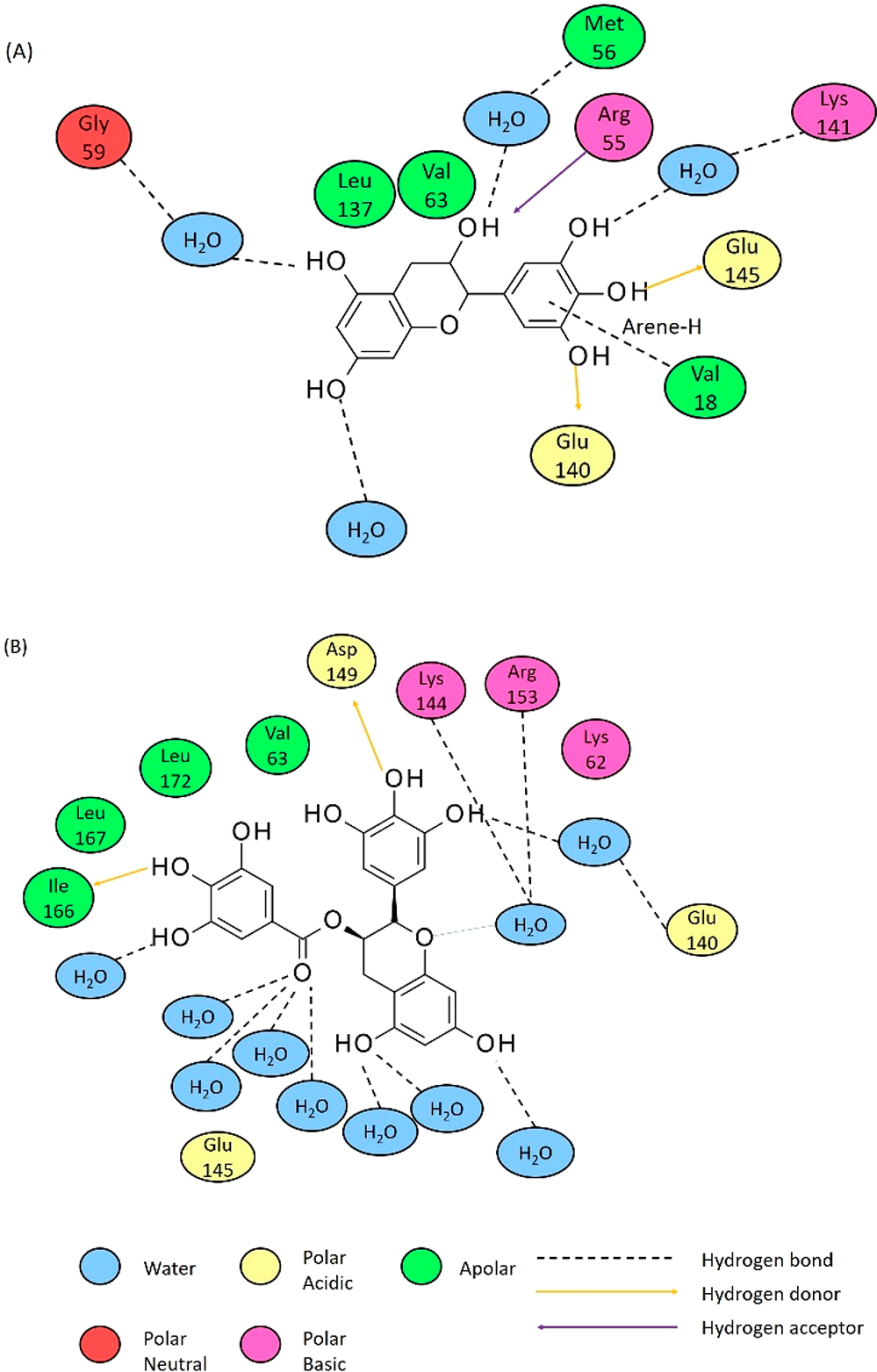
Proposed binding regions of the RAD52 inhibitors: (A) **63** and (B) **64**. Adapted from eLIFE, 2016, Vol. 5, e14740, Sarah R. Hengel, Eva Malacaria, Laura Folly da Silva Constantino, Fletcher E. Bain, Andrea Diaz, Brandon G. Koch, Liping Yu, Meng Wu, Pietro Pichierri, M. Ashley Spies, Maria Spies, Small-molecule inhibitors identify the RAD52-ssDNA interaction as critical for recovery from replication stress and for survival of BRCA2 deficient cells10.7554/eLife.14740PMC498276027434671,^169^ with use of the Attribution 4.0 International (CC BY 4.0) open access license.

Hengel et al. embarked on a discovery program for small molecule inhibitors of RAD52, through use of a HTS of the MicroSource SPECTRUM collection.^169^ Initial hits were tested for RAD52 binding in FRET-based assays, giving five hits that showed an IC_50_ in or near the nanomolar range. Of these hits, two compounds, (−)-epigallocatechin (**63**, Figure 22) (RAD52 DNA binding IC_50_ = 1.8 ± 0.1 μM) and epigallocatechin-3-monogallate (**64**, Figure 22) (RAD52 DNA binding IC_50_ = 277 ± 22 nM), were shown to physically bind to RAD52 by NMR and were shown to prevent binding and annealing to RPA-coated ssDNA. Virtual screens of **63** and **64** within the RAD52 ssDNA binding groove seem to indicate that these compounds show binding in this region. Notable interactions include Arg55, Lys65, Arg153, and Arg156 in the vicinity of **63** and **64**, which when docked have previously been shown to impact ssDNA binding. Additionally Lys141 and Lys144 have been shown to be important previously in RAD52 function in yeast and are thought to be somewhat evolutionarily conserved; some of these binding interactions are schematically reported in Figure 23. **63** and **64** were shown to dysregulate DSB repair in BRCA1/2-depleted and MUS81-depleted cells. Furthermore, both compounds showed activity in killing BRCA1/2-depleted cells in addition to MUS81-depleted cells. This phenomenon of lower cell viability in MUS81-deficient cells has been reported previously.^170^ By use of **63** and **64**’s predicted docking models, an *in silico* screen was embarked upon, using the AnalytiCon Discovery MEGx Natural Products Screen Library, which is made up of natural products from plants, fungal, and antimicrobial sources. From this library, NP-004255 (**65**, Figure 22) (RAD52 DNA binding IC_50_ = 1.5 ± 0.2 μM) was identified, which had the same binding mode as **63** and **64**. **65** underwent the same binding analysis as **63** and **64**, where it was shown to bind to RAD52 and RPA and to inhibit the binding of RAD52 to ssDNA in addition to the ssDNA–RPA complex. However, **65** did not affect RAD52’s binding to dsDNA or affect DNA binding by RPA.

**Figure 24:**
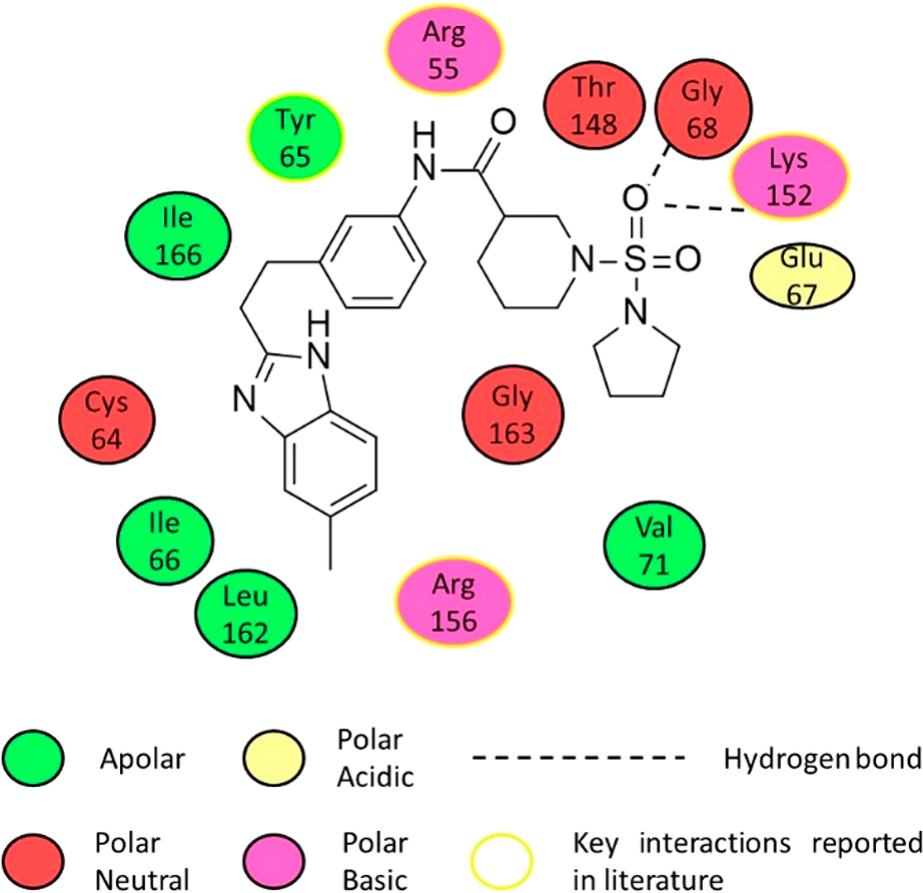
Proposed binding regions of **66**. Reproduced with permission of The Royal Society of Chemistry, from LiJ.; YangQ.; ZhangY.; HuangK.; SunR.; ZhaoQ.Compound F779-0434 causes synthetic lethality in BRCA2-deficient cancer cells by disrupting RAD52–ssDNA association. RSC Advances, Vol. 8, Issue (34), , pp 18859–1886910.1039/c8ra01919cPMC908061535539677,^171^ Copyright 2018, permission conveyed through Copyright Clearance Center, Inc.

Li et al. investigated new RAD52 inhibitors, beginning with use of a virtual screen, by docking the compounds inside the RAD52 monomer.^171^ This HTS consisted of 47 737 compounds, with the 30 top-performing compounds being sorted into groups of 5 in relation to their chemotype. These 30 compounds were tested for their ADMET properties, and 4 were selected due to their druglikeness for further testing: F779-0434 (**66**), F848-0436 (**67**), G640-1014 (**68**), and D207-0130 (**69**) (Figure 22), with **66** appearing to be the most promising RAD52 inhibitor. Similar to the work conducted by Spies et al., all 4 compounds tested were seen to bind in the ssDNA binding pocket. Key binding interactions were observed between the 4 compounds and Arg 55, Lys 152, Arg 165, and Tyr 65. Notably the interaction with Arg 55 is shared between **61** and **66**, suggesting this is a key RAD52 interaction. These binding interactions can be seen in more detail in Figure 24. **66** was shown to be able to selectivity inhibit growth in a BRCA1/2-dependent pancreas cancer cell to 50% at 10 μM, whereas in the same concentration over 90% of cells from a healthy cell line survived. Furthermore, it was shown to inhibit RAD52 ssDNA annulation beginning at 5 μM, and at 20 μM complete inhibition of the process could be seen. However, the other three compounds selected by the virtual screening did not show as promising an effect in these assays.

To date, no RAD52 inhibitors have entered clinical trials. However, the numerous successes seen in this section, particularly *in vitro*, and the greater wealth of information known about the binding pocket and binding modes suggest that the potential for RAD52 inhibitors to work as SL agents is vast.

## 8 PD-1 and Synthetic Lethality

Since the late 19th century, the application of the immune response in cancer therapy has been theorized;^172^ while it was quite controversial through much of this time, it is now seen as a promising approach to anticancer therapy. One of the targets that has gained interest is programmed cell death protein 1 (PD-1), which is responsible for fine-tuning T-cell function, the maintenance of homeostasis within the immune response, acting as a natural break, and initiating the checkpoint response usually associated with periphery tolerance.^173^ However, in cancer, tumor cells take advantage of this and use the mechanism to suppress and evade the immune response. Therefore, the concept of a checkpoint blockade has been proposed, where inhibition of PD-1 causes reactivation of the immune response toward the cancerous cell, utilizing the body’s defenses to fight cancer. Several PD-1 monoclonal antibodies have been successful in clinical trials, leading to FDA approval for several monoclonal antibodies, namely, nivolumab, pembrolizumab, atezolizumab, avelumab, and durvalumab. In various cancers, anti PD-1 agents are standard of care therapies for many malignancies including melanoma, small-cell lung cancer, non-small-cell lung cancer, colorectal cancer, and many more.

It is known that blocking DNA repair mechanisms increases genetic instability, which in turn increases epitope expression on the cancer cell surface, which is usually immunodominant.^174^ It is therefore possible that this increased genetic instability could make the cancer cells more susceptible to the immune response if heightened, making PD-1’s combination with targets involved in the DNA repair appealing as a potential source of synergism, perhaps even to the extent of synthetic lethality. In fact, some DNA-damage regulators such as PARP have been to seen to cause an increase in PD-1 expression, suggesting a likelihood of synthetic lethality. Due to this, PARP and PD-1 joint inhibition has been explored, with synergism seen in preclinical trials in addition to early stage clinical trials, of which there are more than 30 currently in progress in a wide of range of malignancies.^174^ The potential for PD-1 modulation in combination with other targets is evident throughout this review, with numerous ATR inhibitor clinical trials and trials featuring CDK12 loss of function mutants utilizing PD-1 antibodies. While this approach is still new, with the first work being conducted in 2017,^175^ it could be expanded to other targets mentioned within this review such as RAD51. The theory is that any target which, when modulated, causes a reduction in DNA repair could potentially be more susceptible to T-cells involved in the immune response, once the checkpoint response has been modulated.

In this section, numerous SL targets and strategies have been highlighted. With the rapidly expanding number of known SL partners, it would be impossible to cover them all in one review. The majority of the highlighted discovery programs focus on developing a selective potent modulator of a specific target and work to optimize through a classical medicinal chemistry approach. It is usually at a later stage of the screening cascade where SL is investigated, initially though combination studies in cells; thus, fewer compounds are screened for SL, which renders it difficult to comment on whether or not the SAR approach is in fact increasing SL. This is understandable as, despite being known for many years, SL approaches are still in their infancy; however, the number of promising compounds in late-stage clinical and preclinical studies gives hope that one of these targets will eventually be employed in an SL approach within the clinic.

## 9 Challenges and Opportunity in Developing New SL Drugs

To date, numerous potential synthetic lethal genes have been identified through statistical screening,^176^ genetic screening,^177^ high-throughput phenotypic screening,^178^ and combinational approaches. However, this has yet to correlate with improved medicines in the clinic, with only one SL pair being exploited to date and four drugs acting through the same PARP1 SL mechanism (**1**, **2**, **3**, and **4**). One of the largest barriers to the development of more drugs acting on SL is the heterogeneity of cancer,^179^ with many SL targets only existing in a small number of cancer subtypes. This therefore means sample sizes are dramatically lowered, be they statistical data points or patient samples for *in vitro*/*in vivo* experiments crucial for validating the SL phenotype. Second, more mechanistic detail must be obtained to evaluate how best to drug SL targets. As shown in this review, the mechanistic understanding of many SL relationships is poor, with PARP/BRCA2 being one of the only synthetically lethal gene pairs to have received extensive study. With mechanistic understanding of what drives SL still not fully understood, creative approaches have been employed to investigate this vital piece of the puzzle. Li et al. employed a machine learning approach to discover what “characteristic functional features” are required for a protein-coding gene to have the potential for synthetic lethality.^180^ The methodologies involved are outside the scope of this review; however through this study Li et al. discovered 15 key characteristic functional features, 14 of which had previously been reported to play a role in SL to some degree in the literature (these were given a descriptor and can be seen in Table 6). These descriptors are generated using machine learning algorithms, which through an enrichment system derived from gene ontology terms and the Kyoto encyclopedia of genes and genome pathways represent functional features. It is hoped that this study can be utilized in both computational and experimental methods for selecting potential genes to investigate for SL. Other big data approaches^181^ have been employed to try to aid the “hypothesis driven” methods that have been used to discover SL pairs previously, with a collated and curated list of SL interactions available for free online via BioGrid.^182^

**Table 6:** Key Characteristics Discovered through a Machine Learning Approach Conducted by Li et al.^180^

maximum relevance and minimum redundancy (mRMR) feature name	description of function
GO: 0097505	This term represents the cellular component named RAD6–18 complex: a previous study in *Saccharomyces cerevisiae* confirmed RAD6 and RAD18 exhibit SL. The function in humans is similar to that in yeast, and therefore it is likely that these two are SL in humans.^190^
GO: 0070449	This term represents a cellular component named the elongin complex which consists of the elongin transcription factors elongin A, B, and C. These have been seen previously to be SL with the von Hippel–Lindau protein complex.^191^
GO: 0033503	This term represents the histone H2B ubiquitination complex. It generally modulates heterochromatin-independent histone methylation. The individual components of this complex perform similar and redundant tasks involved in the regulation of cell survival.^192^
GO: 1901136	A catabolic process associated with carbohydrate derivation, this consists of chemical reactions that contribute to the digestion and breakdown of carbohydrates. The relationship between catabolic processes for carbohydrates and SL has been previously reported, with genes like BCL-2 being involved both in cancer and in carbohydrate digestion.^193^
GO: 0008234	A molecular function involving cytosine protease and thiol protease activity that plays a role in oncogene addiction and is involved in the maintenance of cell viability suggesting a potential for SL.^194^
GO: 1903772	A complex involved in the regulation of viral budding. Genes involved in the formation of this complex such as SKD1, LIP5, and IST1-LIKE1 are involved in specific SL mechanisms.^195^
GO: 0003383	The biological process of the actin-mediated contraction of the apical end of a polarized epithelial cell. Recently it was discovered that Lin-44 and Wnt participate in SL processes in addition to regulating apical constriction; therefore it is theorized that dysfunctional apical constriction could lead to SL.^196^
GO: 1903333	This describes the negative regulation of protein folding: it is theorized that this may cause toxic intermediates under the control of chaperones that may cause further cell death.^197^
GO: 0070202	This describes the regulation of protein localization in chromosomes. Two groups of genes, which encode for either the structural maintenance of chromosome proteins or chromosome stability proteins including BRCA, show the SL phenotype.^198^
GO: 0070087	A biological process of chromoshadow domain binding is predicted to be involved with SL, as the specific heterodomain protein HP1γ, which is involved in SL, is predicted to bind at the chromoshadow domain, thus connecting this domain to cell viability.^199^
GO: 0004883	This feature describes the activity of a glucocorticoid receptor or the participation in transmission and reaction of a glucocorticoid. Recent research shows p53 and GRα/ß are involved in these biological functions and are SL in non-small-cell lung cancer.^200^
GO: 2001034	The biological process of DNA repair through HR or n-HEJ, such as PARP.^19^
GO: 0000209	The process of protein polyubiquitination; two examples of genes involved in this process are Slx5 and Slx8, which have also been reported to show synthetic lethality with each other. This suggests a connection between protein polyubiquitination and SL.^201^
Hsa04612	This represents antigen processing and presentation in the human immune response. Two genes indicated in this response, BRAF and NRAS, have been seen to be synthetically lethal with each other in melanoma.^202^

The ability to generate compounds capable of induced small-molecule synthetic lethality can be hampered by the complexity and size of the binding regions of the targets associated with SL. One such example noted earlier in the text is the attempted induction of SL through the blocking of RAD51–BRCA2 interaction; this is a protein–protein interaction that has in the past been difficult to modulate with small molecules, due to the size of the compound in comparison to the large binding region between the proteins. Work performed earlier in the decade by Hyvönen et al. attempted to modulate the RAD51–BRCA2 interaction through a fragment-based approach. Through protein engineering of RADA, the RAD51 analogue present in *archaea*, a monomeric form of RAD51 was able to be synthesized.^183^ A fragment-based approach in 2013 demonstrated, through use of isothermal titration calorimetry (ITC), NMR, and X-ray crystallography, the first small molecule binding at the FxxA site of BRC4, a key binding region for the protein–protein interaction between RAD51 and BRCA2. However, a low success rate of only 0.2% was seen in the ITC assay in comparison to previous enzymatic assays. Two fragments were identified from this screen with low mM affinity; while low, it was a good starting point for further evaluation. This work was expanded upon in 2015, resulting in a 500-fold increase in affinity from the initial hits. Although the most promising hits from this series were unable to be crystallized, they performed well in the ITC assay, which seemed to indicate binding at the same site.^184^ Taken together, this fragment-based approach, utilizing biophysical assays such as ITC, NMR, SPR, and X-ray crystallography, demonstrates another possible tool in the pursuit of challenging SL targets. The genetic screening approach to evaluate possible SL pairs has gained new tools since the turn of the century. Previously, this was achieved through use of RNA interference (RNAi), where small-interfering RNAs (siRNAs) and/or short hairpin RNAs (shRNA) were employed to knockout genes to investigate whether the simultaneous knockout of two genes caused SL. This has been achieved in a high-throughput manner. An example of such is Novartis’s DRIVE program,^177^ which screened 7837 genes in 398 cell types. Numerous potential SL relationships were discovered in these studies, and these data could serve as a valuable tool for future drug-discovery programs, as all Novartis’s data on the DRIVE project is freely available through an online portal (https://oncologynibr.shinyapps.io/drive/). However, RNAi-based studies tend to suffer from poor reproducibility and comparison between studies, which can result in false positives and an inability to distinguish between on-target and off-target effects. Recent years have also seen the development of CRISPR-CAS9, which is functionally similar to RNAi but offers a higher-degree of reproducibility. This method involves a strand of single guided RNA (sgRNA) that allows the CAS9 endonuclease to bind with a sequence specific to that strand of sgRNA, thus enabling further modulation or regulation.^185^ Its advantages over RNAi are the following: it depletes genes in a more consistent manner; it has a higher degree of sensitivity with genes that have low levels of mRNA or short mRNA half-lives; it reduced RNA interference from heterogeneity among different cell lines.^186^ This technique has been employed in studies investigating synthetic lethality. As an example, Wang et al.^187^ performed CRISPR analysis in 3 different cell lines that had been treated by the ATR inhibitor, **24**, in order to discover more SL pairs for ATR. From these studies, it was discovered that RNASEH2 was SL with ATR inhibition *in vitro* and *in vivo*. RNASEH2 is rarely upregulated in cancer subtypes; however this was in fact observed in prostate adenocarcinoma, suggesting a potential new biomarker that could be explored in this disease area. The same CRISPR-CAS9 studies reported that ATR inhibitors were SL with numerous other genes involved in the ATR pathway, suggesting that the whole ATR pathway is critical for cell survival; furthermore, members from the BRCA family were observed to be synthetic lethal with ATR, showing potential SL between ATR and HR-mediating targets. Overall, this demonstrates the potential of the CRISPR-CAS9 technique to be employed in the discovery of previously unknown targets for SL and guides further research. Furthermore, CRISPR-CAS9 in theory could be employed for treatment of cancers. This field is quite controversial with many ethical dilemmas that need to be addressed; however to date, a number of CRISPR-CAS9 based therapies have entered clinical trials.^188^ Should these be successful, it is possible in the future that CRISPR-CAS9 could be used in a therapeutic way to trigger SL.

Testing SL using *in vitro* models can be difficult. The biological models that are used for the development of such molecules usually include pairs of isogenic cell-based models. These are not an entirely accurate prediction of potential SL phenotypes, as it has been shown that SL pairs can be dependent on context specificity, with cancer presenting a vast array of genotypes. Small differences in the genotype can mean that a pair of small molecules modulating an SL interaction may be effective in one circumstance but not another. Examples of this phenomenon can be seen in colorectal cancer, where the presence of KRAS and NRAS mutations is a predictor for resistance to anti-EGFR antibodies.^189^ This is already being used in the clinic and demonstrates the importance of understanding the impact of context specificity on the application of SL-dependent treatments.

As it has been shown in the regulatory body approval of the PARP inhibitors, these drugs can be very effective in treating tumors with “BRCAness”, i.e., a tumor where the patient shows impeded function of one of the BRCA family genes involved in HR.^203^ Unfortunately, while a number of cancers (e.g., ovarian cancer)^204^ feature the BRCAness trait, the ability to administer a single inhibitor to induce SL is diminished in cancers that do not express BRCAness. To have these compounds truly be effective and initiate small-molecule-induced synthetic lethality, modulators must be administered in combination with one another in order to see a synergistic effect.^147^ This has been demonstrated previously with use of the PARP1 inhibitor **2** and temozolomide, an orally bioavailable monofunctional DNA-alkylating agent.^205^ The use of combinational therapy can be challenging, as the number of therapeutics in combination increases the likelihood of unwanted side effects, in addition to the potential for unknown drug–drug interactions.^206^ In order to minimize these issues, it is vitally important that the mechanism of action of the compounds and their safety profiles be well understood. If this is the case, the potential for combinational treatment is vast. Work has been conducted into evaluating more potential drug combinations that currently exist by Heinzel et al.^207^ Through a bioinformatics approach, they investigated 358 ovarian cancer trials utilizing the clinicaltrials.gov Web site. The search was refined further by use of keywords, eventually resulting in 68 trials that showed 61 unique drug interactions. These data allowed them to identify numerous combinations that are not currently being investigated in clinical trials but have the potential for SL and merit further investigation. These data illustrate that while combination treatments present challenges, the wealth of data on some of these well-known compounds in several clinical trials can be used as an asset, and there are great opportunities that can still be explored.

It is hypothesized by the authors that the use of small molecule-induced synthetic lethality should be approached with caution. In traditional SL approaches, where one of the genes lack function, i.e., through mutation in certain cancers, use of an inhibitor for the other SL gene targets the cancer cell and leaves the healthy cell employing a somewhat selective therapy. However, if this approach is replicated with small molecules, it is possible that the mechanisms these genes perform in DNA repair are utilized throughout healthy cells in addition to cancerous cells. Therefore, while cancer cells are more rapidly dividing and therefore will be reliant on HR, noncancerous cells will also use these mechanisms to some degree, resulting in unwanted side effects. Indeed, cancer cells are less genetically stable than healthy cells and therefore feature more DNA breaks, thus being more reliant on HR. This likely confers to small molecule-induced synthetic lethality a degree of selectivity for cancerous cells over healthy ones. The implications for this approach to be employed safely are that the underlying biology of the targets in healthy and cancer cells should be fully understood so as to apply this innovative approach to suitable target pairs.

Synthetic lethality, as shown throughout this review, has been primarily employed in oncology, attempting to disrupt the survival pathways of cancerous cells; however, this is not the only potential application of synthetically lethal therapies. The discovery of new antibiotics is an urgent need, as strains of resistant bacteria such as methicillin-resistant *Staphylococcus aureus* (MRSA) continue to be a major risk of infection in hospitals throughout the world.^208^ Due to bacteria being relatively simple organisms, they are reliant on HR for DNA damage repair;^209^ therefore SL has been looked at as a possible way of developing antibacterials. Charusanti et al. utilized a systems biology-guided approach to discover SL pairs for bacteria.^210^ The SL pairs identified from the virtual screens employed in these studies were confirmed experimentally with a precision rate of 25–43%. A virtual screen of compounds that were able to dock with 4 pairs of targets was performed subsequently. Two pairs of targets were found through their studies to be synthetically lethal (hemF/hemN, lpdA/sucC), one pair where both genes were found to be singularly essential (glyA/serA), and one pair where only one of the genes was known to be singularly essential (mdh/ppc). These studies did not yield compounds capable of inducing synthetic lethality; however the ability to identify SL pairs in bacteria opens the door to focused drug-discovery programs that may have more success.

Furthermore, additional potential applications can be seen in antiparasitics to treat diseases such as drug-resistant malaria. Due to the high level of conservation of genome integrity and cell cycle genes between species, it has been possible to conduct large screens in yeast and then use the SL pairs discovered to apply to other organisms, using the analogue genes in for example *plasmodium*.^211^ Using this approach, Lee et al. were able to compare yeast SL genes to their analogues present in the malaria parasites *Plasmodium falciparum* and *Plasmodium vivax* for the potential SL treatment of malaria in humans. Genes were selected that do not have an analogue in mammals, so as to minimize the chance of cross-species promiscuity. Through these studies five SL partners in the *plasmodium* were discovered. These findings warranted further investigation. To aid this, the group identified a number of commercially available compounds that already act upon these targets, showing a potential application of SL in antiparasitics. Many of the SL relationships discussed in this text have analogues in bacteria and parasites that play key roles in HR due to a high degree of evolutionary conservation. These examples, such as RAD51’s analogue in bacteria RecA or TbRAD51 in *T. brucei*, perform similar roles in their associated organisms and are heavily involved in HR and DNA repair.^212,213^ Of course, selectivity between species would be vitally important should any of these targets be explored, which could be challenging due to the large degree of homology between them; however, if this selectivity issue could be resolved, it could expand the usefulness of drugs that function through an SL mechanism greatly and warrants further exploration within the field.

## 10 Future Prospects and Final Thoughts

It has clearly emerged that the field of SL has much potential for oncology in a need that the personalized medicine from the previous 20 years has struggled to fill. SL offers a way to treat cancer that, in theory, leaves healthy cells unaffected and therefore has reduced side effects compared to nonspecific chemotherapy. However, the application of SL modulators still has a long way to go, as not all cancer subtypes have clearly defined deficiency, such as has been seen with the application of PARP1 and BRCA1/2 inhibitors or the 29 clinical trials currently involving ATR inhibitors. As the fields’ understanding of the underlying cancer biology increases, in addition to the growing network of tools to discover more SL pairs and, importantly, the mechanisms involved in SL, the viability of SL related drugs will increase. For this to occur, cross-disciplinary communication is vital, from target selection to compound design and optimization and beyond into *in vitro*/*in vivo* and into the clinic, with a willingness to risk and explore previously uninvestigated targets. Furthermore, the way in which SL drugs are implemented will be key to their success. For example, if full small molecule-induced synthetic lethality is to be used, i.e., inhibiting two SL pairs selectively at once, one must consider the greater potential for resistance as the cancer cell survival pathways adapt. Either it must be accepted that these compounds have a short window of effectiveness, or these mechanisms of resistance must be studied and anticipated so that further treatment can be administered to restore sensitivity, as is attempting to be achieved with PARP1 resistance through the restoration of function of BRCA1/2 using RAD51-BRCA2 inhibitors.^143^ Alternatively, if SL compounds can be implemented to impede HR in cancers that have undergone DNA stress, such as alkylation agents, nonspecific chemotherapeutics, or ionization, it is possible that through this method SL could be employed to lessen the dosage and therefore side effects of these treatments, with a reduced risk of acquired resistance from the SL pathway. Additionally, it has been theorized that SL drugs would be most effective in early stage cancers, as it is hypothesized that premalignant cancers are less heterogenic, and therefore this is where SL is likely to have the greatest impact.^214^ Obviously, for this to be viable, early detection of cancers in the clinic has to be achievable, and this chemopreventive approach is very much still in its infancy, existing primarily in animal models.^215^ With these considerations in place, it can be seen that the future for SL modulations could be bright, and in years to come the number of projects shining light on this interesting method of combating cancer will surely grow, and hopefully this will be reflected in the clinic. We hope this work inspires the medicinal chemistry community to develop novel compounds that can be exploited to discover innovative SL pathways in a chemical biology framework and, through utilization in drug discovery programs, identify SL-based anticancer compounds.

## Supporting Information Available

Molecular formula string (CSV)

## Abbreviations Used

ATRi: ATR inhibitor
AZ: AstraZeneca
BER: base excision repair
BTK: Bruton’s tyrosine kinase
CL: clearance
CtIP: CTBP interacting protein
CYP3A4: cytochrome P450 3A4
*D*_abs_: maximum absorbable dose
DNA-PKi: DNA-PK inhibitor
DSB: double-stranded break
HER2: human epidermal growth factor 2
HR: homologous recombination
HTS: high-throughput screen
ITC: isothermal titration calorimetry
MMEJ: microhomology-mediated end joining
MRSA: methicillin-resistant *Staphylococcus aureus*
NER: nucleotide excision repair
NHEJ: nonhomologous end joining
PARP: poly (ADP-ribose) polymerase
PARPi: PARP inhibitor
RAD52i: RAD52 inhibitor
RNAi: RNA interference
RPAi: RPA inhibitor
sgRNA: single-guided RNA
shRNA: short hairpin RNA
siRNA: small-interfering RNA
SL: synthetic lethality
SSB: single-stranded break
ssDNA: single-stranded DNA
*V*_ss_: volume of distribution
WT: wild-type
